# Effects of working memory and reward reactivity on externalizing behaviors in the ABCD study

**DOI:** 10.1016/j.dcn.2026.101743

**Published:** 2026-05-25

**Authors:** Elana Schettini, Jennifer Cheavens, Zeynep M. Saygin

**Affiliations:** a Department of Psychiatry, VA Connecticut, West Haven, CT, USA; b Department of Psychiatry, Yale School of Medicine, New Haven, CT, USA; c Department of Psychology, The Ohio State University, Columbus, OH, USA; d Center for Cognitive and Behavioral Brain Imaging, The Ohio State University, Columbus, OH, USA

**Keywords:** Externalizing behavior, Adolescence, Working memory, Reward, Stressful life events, Functional variability, ABCD study

## Abstract

Deficits in working memory and reward reactivity may contribute to behavioral dysregulation. Using behavioral and neural data from the Adolescent Brain and Cognitive Development (ABCD 5.1) study, we examined associations among biological systems, environmental risk, and childhood externalizing behaviors (i.e., aggression, rule breaking). Behavioral measures included List Sorting and Cash Choice tasks to assess working memory and reward processing, respectively. Neural measures included the functional magnetic resonance imaging (fMRI) emotional n-back (EN-back) and Monetary Incentive Delay (MID) tasks to assess working memory (2-back vs. 0-back) and reward processing (anticipation: reward vs. neutral; feedback: reward success vs. failure), respectively. We conducted linear mixed effects models with discovery and replication samples to investigate correlates of externalizing behaviors at baseline (behavioral: *n* = 7448; neural: *n* = 3265) and two years later (behavioral: *n* = 7127; neural: *n* = 3151). We also tested whether these relationships differed by sex, adjusting for age, socioeconomic status, and stressful life events. Lower behavioral working memory was related to concurrent externalizing behaviors. Baseline stressful life events was the strongest predictor of externalizing behaviors two years later, highlighting environmental adversity’s role in development of externalizing behavior. Reward reactivity (behavioral or reward anticipation and feedback neural activation) and working memory neural activation were not associated with externalizing behaviors at either time point. Findings suggest that environmental stress and behavioral working memory explain variability in externalizing behaviors within the scope of the measures examined here. These findings highlight the need for continued research clarifying the neural mechanisms underlying externalizing behavior.

## Highlights

• Worse short-term memory is associated with more aggression & rule-breaking
• Greater early life stress correlates with more aggression & rule-breaking
• Stressful life events predict increases in externalizing behaviors 2 years later
• Substantial variability in brain activation during working memory & reward fMRI tasks

## Graphical Abstract

**Figure:**
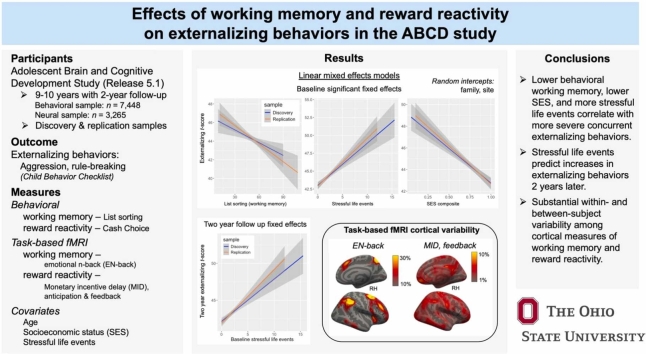

## 1 Introduction

Externalizing behaviors characterize a broad-spectrum psychopathology domain comprised of impulsive behaviors, such as aggression and rule breaking. When these behaviors emerge during childhood, they can become more entrenched as biological vulnerabilities interact with environmental risk factors throughout development (e.g., see Beauchaine and McNulty, 2013; Shaw, Hyde, & Brennan, 2012). Therefore, early identification of children at risk for escalating externalizing behaviors is crucial for implementing timely interventions with the potential to alter developmental trajectories. The ontogenic processes model of externalizing psychopathology (Beauchaine and McNulty, 2013) proposed a common developmental trajectory for severe externalizing behaviors (e.g., high antisociality, substance use disorders). This model suggestd that trait impulsivity is an underlying vulnerability to externalizing spectrum disorders (ESDs; i.e., ADHD, oppositional defiance disorder, conduct disorder, substance use disorders, and antisocial personality disorder) that is expressed age-appropriately across the lifespan and varies depending on interactions between biological vulnerabilities and environmental risk factors (Beauchaine et al., 2014).

According to this model, deficient meso-limbic dopamine function reduces reward reactivity among individuals with ESDs, eliciting reward-seeking behaviors characteristic of trait impulsivity (Zisner and Beauchaine, 2016). Further, comorbid deficiencies in meso-cortical dopamine function (behaviorally expressed as poor cognitive control, or a lower capability to engage in goal-directed behavior) further increases risk of mood lability. The ontogenic processes model for ESDs also highlighted the influence of environmental risk factors in exacerbating risk for ESDs throughout development. Thus, it is possible that accounting for low reward reactivity and poor cognitive control, in addition to environmental experiences, will enhance identification of risk for problematic externalizing behaviors.

Neural reward processing is thought to be supported by the cortico-striatal-thalamic loop, which involves interconnected subcortical and frontal cortical regions. Authors have shown that this neural loop reacts to substance- and food-related rewards in both animal (Haber and Knutson, 2010, Ikemoto, 2010) and human (Kalivas and Volkow, 2005, Kelley et al., 2005, Weiss, 2005) paradigms. Altered reward processing has been observed in individuals with ESDs. Behaviorally, children with ADHD exhibited worse performance on motivational tasks, and in girls, these effects intensified over time (Skogli et al., 2017). Neuroimaging studies showed abnormal activity within the cortico-striatal-thalamic loop among individuals with ADHD, conduct disorder, and substance use disorders (Holz et al., 2017, Luijten et al., 2017, Plichta and Scheres, 2014). Thus, activation in the cortico-striatal-thalamic loop seems to reflect differences in subjective valuation and may enhance prediction of externalizing behaviors.

Deficient cognitive control has often been considered a transdiagnostic risk factor for psychopathology (e.g., Abramovitch et al., 2021). Individuals with ESDs demonstrated poorer cognitive performance across executive function tasks (Arnett et al., 2022, Skogli et al., 2017), reduced parasympathetic regulatory capacity (Zhang et al., 2020), and altered neural reactivity during task-based imaging (Karlsgodt et al., 2018, Moeller et al., 2014). The fronto-parietal multiple demand (MD) network supports domain-general processing of cognitive control (Assem et al., 2020, Duncan, 2010) and was reliably activated by working memory task-dependent functional magnetic resonance imaging (fMRI) measures in both adults and children (Fedorenko et al., 2013, Satterthwaite et al., 2013, Schettini et al., 2023). Importantly, children's neural selectivity within the MD network positively correlated with their concurrent task performance on working memory tasks (Satterthwaite et al., 2013, Schettini et al., 2023), suggesting that neural selectivity of the MD network reflects ongoing cognitive effort during working memory fMRI tasks. Thus, working memory, one of the three components of cognitive control (Friedman and Miyake, 2017, Miyake and Friedman, 2012), appears to adequately capture individual variability in domain-general cognitive control capacity. Given that externalizing behaviors are thought to arise, at least in part, from deficient top-down regulation of impulsive tendencies, it is plausible that greater externalizing severity would also correlate with neural markers of working memory.

Prior behavioral studies showed that an individual’s valuation of expected rewards influenced their allocation of cognitive effort (Strang and Pollak, 2014, Dixon and Christoff, 2012). In addition to behavioral evidence that the reward and cognitive control systems interact, the underlying neural systems are structurally and functionally intertwined (Haber, 2016; Salehinejad et al., 2021). Functional neural reactivity was associated with expected reward-values (Alexander and Brown, 2011, Dixon and Christoff, 2012) and was moderated by outcome certainty (Satterthwaite et al., 2007), further suggesting that allocation of cognitive resources varies based on motivational demands. Only one study has investigated the interaction effect between reward and cognitive control in relation to neural representations of externalizing behaviors (Rodriguez-Thompson et al., 2020); the authors showed that adolescents with externalizing behaviors exhibited greater frontal activation than typically developing adolescents during previously rewarded response inhibition trials, but no differences during trials without reward. These findings emphasize the importance of modeling functional interactions to better understand the neural underpinnings of externalizing behavior. Because cognitive engagement appears susceptible to changes in reward contingencies, further investigation of interactions between working memory and reward reactivity are warranted.

Sex-related differences are also evident throughout development and should be considered in models examining developmental trajectories of externalizing behavior. Cognitive control performance was associated with different neural correlates for girls and boys, despite comparable overall performance (Cuevas et al., 2016, Lissek et al., 2007), suggesting that underlying brain mechanisms supporting efficient functioning may differ between sexes. Consistent with this speculation, sex showed significant moderation effects on brain-behavior relationships observed in depressed (Jenkins et al., 2018, Wang et al., 2023) and externalizing (Beauchaine et al., 2008, Ducharme et al., 2011) samples, highlighting the importance of explicitly modeling sex interactions in this area of research.

In the present study, we leveraged data from the Adolescent Brain and Cognitive Development (ABCD) study to examine the relationship between externalizing behaviors and measures of reward processing and working memory. Behavioral assessment of working memory and reward were measured with the National Institutes of Health (NIH) Toolbox List Sorting and Cash Choice tasks, respectively; neural assessment of working memory and reward were measured with the emotional n-back (EN-back) and monetary incentive delay (MID) fMRI tasks, respectively. We tested interactions between sex, working memory, and reward, including a three-way interaction among these factors. All models controlled for age, socioeconomic status (SES), and stressful life events, given their proposed role as risk factors for ESDs (Beauchaine and McNulty, 2013). To address concerns about poor reliability of fMRI in large-scale studies (Elliott et al., 2020, Noble et al., 2021) and the resulting loss in power to detect brain-behavior relationships in the ABCD study (Gell et al., 2024, Kennedy et al., 2022), we used a discovery and replication sample and report within- and between-subject variability for neural measures. We predicted that poorer working memory (i.e., performance and neural activation), behavioral preference for immediate vs. delayed reward, and blunted neural activation to reward (i.e., anticipation and feedback) would be associated with more severe externalizing behaviors at baseline and two years later; we also predicted that these relationships would differ by sex and that stronger working memory capacity would be protective against reward-related vulnerabilities.

## 2 Method

### Participants

**Fig. 1:**
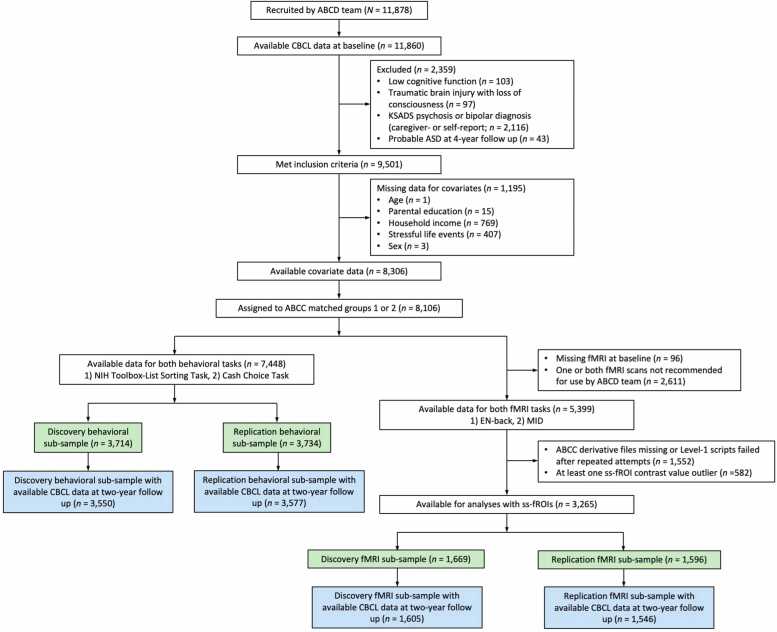
Consort diagram depicting levels of inclusion and exclusion criteria. *Note*. Participant flow for behavioral and imaging sub-samples; final baseline sub-samples are highlighted in green, and final two-year follow-up sub-samples are highlighted in blue. ABCD = Adolescent Brain and Cognitive Development study; CBCL = Child Behavior Checklist; KSADS = Kiddie Schedule for Affective Disorders and Schizophrenia; ASD = Autism Spectrum Disorder; ABCC = ABCD-BIDS Community Collection; EN-back = emotional n-back; MID = Monetary Incentive Delay; ss-fROI = subject-specific functional region of interest.

The sample was a subset of participants drawn from the ABCD dataset, which is an ongoing multi-site study examining brain and cognitive development across adolescence and, eventually, early adulthood (Volkow et al., 2018). The ABCD team recruited 11,878 participants, aged 9–10 years (Garavan et al., 2018), who completed a battery of behavioral, sociocultural, and wellness measures annually and were scanned every two years (https://abcdstudy.org/scientists/). The present study included participants with available baseline Child Behavior Checklist (CBCL; Achenbach et al., 2009) data; a consort diagram (Fig. 1) depicts the participant flow regarding inclusion in final analyses. Participants were excluded if they exhibited low cognitive function (NIH-Toolbox Total Cognition Fully-Corrected composite score >2 standard deviations below group mean), experienced a traumatic brain injury with loss of consciousness, met criteria for bipolar or psychotic disorder at baseline (caregiver- or participant-report), or met criteria for probable autism spectrum disorder (ASD) diagnosis at their 4-year follow-up (ASD was not assessed at baseline or 2-year follow-up).

#### Discovery and replication samples

We used discovery and replication samples generated by the ABCD-BIDS Community Collection (ABCC; Feczko et al., 2021) team. ABCC houses community-shared ABCD neuroimaging data that conforms to the pre-determined quality control standards of the Brain Imaging Data Structure (BIDS). The ABCC discovery and replication samples were drawn from the full ABCD sample and were matched on site, age, sex, ethnicity, grade, highest level of parental education, handedness, combined family income, exposure to anesthesia, and family-relatedness; for more information about these matched samples, please refer to the ABCC’s recommendations webpage (https://nda-abcd-collection-3165.readthedocs.io/latest/recommendations/). Eligible participants who were not assigned to matched groups 1 or 2 by the ABCC team were not included in analyses.

#### Behavioral vs. imaging sub-samples

Behavioral analyses included the subset of the final sample that had available NIH Toolbox – List Sorting task and Cash Choice task data (*n* = 7448; Fig. 1 depicts consort diagram). Neural analyses included the subset of the final sample who had available subject-specific fMRI data for both task-dependent paradigms (*n* = 3265; Fig. 1 depicts consort diagram). See Table 1 for demographics of the final baseline discovery and replication samples.

**Table 1:** Demographics of the baseline samples.

	Behavioral sub-sample		fMRI sub-sample	
	Discovery	Replication	Discovery	Replication
	*n* = 3714	*n* = 3734	*n* = 1669	*n* = 1596
	*M*(*SD*) or %	*M*(*SD*) or %	*M*(*SD*) or %	*M*(*SD*) or %
Age (years)	9.92(.62)	9.91(.62)	9.97(.62)	9.98(.63)
Sex (% male)	50.13^a^	52.70^a^	48.29^b^	52.01^b^
Handedness (% right dominant)	80.61	80.10	81.36	83.58
NIH Toolbox, total cognitive function t-score, fully corrected	48.59(10.95)	48.71(11.00)	49.44(10.55)	49.74(10.79)
Race/Ethnicity (%)				
White	58.51	57.07	62.57	61.79
Black	11.11	11.80	7.20	8.16
Hispanic	18.87	18.66	18.60	16.31
Asian	2.18	2.33	1.98	2.20
Other reported	9.33	10.14	9.66	11.54
Household income (%)				
< $50,000	25.01	25.98	22.29	18.98
$50,000-$100,000	29.08	29.46	28.94	31.77
> $100,000	45.91	44.56	48.77	49.25
Highest parent education (%)				
No high school diploma	4.79	4.50	3.59	2.82
High school diploma or equivalent	23.07	24.99	21.21	22.37
College graduate	43.30	41.51	46.73	44.30
Graduate or Professional degree	28.84	29.00	28.46	30.51

*Note*. Demographics of the baseline samples included in behavioral and neural fMRI analyses separated into discovery and replication samples. After exclusions, there was a significantly higher proportion of male participants in the replication sample compared to the discovery sample in both the behavioral and fMRI samples (^a^ χ^2^(1) = 4.82, *p*-value = 0.028; ^b^ χ^2^(1) = 4.35, *p*-value = 0.037).

As only 205 participants with usable fMRI data had missing values on one or both of the behavioral tasks, the fMRI sample in Table 1 is largely representative of the sample included in analyses with both behavioral and neural measures of working memory and reward. To estimate future externalizing behavior, we examined a large subset of participants whose caregivers completed the CBCL at the two-year follow up assessment (Fig. 1 depicts consort diagram).

#### Missing data

Due to the large sample size, no imputation methods were used; participants with missing data for any covariate (i.e., age, parental education, household income, stressful life events) were not included in analyses (Fig. 1 depicts consort diagram). Additionally, because analyses were conducted separately by sex, participants who refused to answer or did not endorse either “male” or “female” were not included in analyses.

### Participant- and Caregiver-Report measures

#### Demographics

Participant age, sex, race, caregivers’ education, income, and other demographic characteristics were reported by a caregiver using a modified version of the PhenX demographic questionnaire (see Barch et al., 2018; Stover et al., 2010). Demographics were obtained from the ABCC participant database (https://nda.nih.gov/edit_collection.html?id=3165).

#### Child behavior checklist (CBCL)

ABCD used a computerized version of the CBCL, a standardized caregiver-report measure of behaviors for children aged 8–18 years (Achenbach, 2009), to assess broad-band externalizing and internalizing psychopathology dimensions. The CBCL included normalized estimates (by sex, age, informant and ethnicity) for a range of psychopathological behaviors. Externalizing *t*-scores from the CBCL were used as the primary outcome variable in this study.

#### Kiddie schedule for affective disorders and schizophrenia (K-SADS)

A computerized version of the K-SADS, a semi-structured assessment based on the criteria listed within the Diagnostic and Statistical Manual of Mental Disorders, Fifth Edition (DSM-5; American Psychiatric Association, 2013), was used to assess symptoms of psychiatric disorders in children (Kaufman et al., 1997, Townsend et al., 2020)*.* The self-administered caregiver-report (on youth) and youth-report (on self) have demonstrated good to excellent agreement with clinician-administered interviews (Kobak and Kaufman, 2015, Kobak et al., 2013); notably, the youth-report was validated on children 12 years and older. The computerized version of the K-SADS used in the ABCD sample was modified (e.g., changes to the wording, tailoring of diagnostic algorithms) to allow for more customizable administration. Although K-SADS items are typically recorded on four-point scales, ABCD data were dichotomized (absent vs. present).

#### Edinburgh handedness inventory

A brief version of the Edinburgh Handedness Inventory was used to assess children’s handedness (Veale, 2014). Children reported which hand they typically use for writing, throwing, using a spoon, and using a toothbrush (5-point scale: always right hand, usually right, both, usually left, always left); the summed scores were used to determine right, left, or ambidextrous handedness.

#### Ohio State University traumatic brain injury (TBI) screen-short version

The Ohio State University TBI Screen was adapted for the ABCD Study as a caregiver-report measure of children’s histories of brain injuries and concussions (Barch et al., 2018)*.* The original measure has shown good test-retest reliability and validity for participant-reported TBI (Corrigan and Bogner, 2007). Children whose caregivers reported TBI with loss of consciousness were excluded from analyses.

#### Adverse life events scale

At the 1-year follow-up, the ABCD team began collecting caregiver- and participant-reports of the Adverse Life Events Scale (Tiet et al., 1998). Participants and caregivers indicated whether 25 events had ever occurred for the participant, and whether it occurred within the past year. Because a measure of stressful life events was not collected at baseline, our team assumed if an event was present but did not occur within the past year at 1-year follow-up, it was present at baseline. We generated a mean composite score for caregiver- and participant-reported adverse life events from the total summed scores (i.e., each event coded as present or absent at baseline).

### Behavioral tasks

#### NIH toolbox – cognition battery

The NIH Cognitive Toolbox is a normalized and well-validated behavioral battery of tasks that assess a range of cognitive processes for native English (Casaletto et al., 2015) and Spanish (Casaletto et al., 2016) speakers. Luciana and colleagues (2018) described each of the seven tasks administered to the ABCD participants, including attention, working memory, cognitive flexibility, reading ability, processing speed, visuospatial memory, and language. Each of the three composite scores (i.e., crystallized, fluid, overall) have shown adequate-to-excellent reliability and validity among children and adults (Akshoomoff et al., 2013; Heaton et al., 2014)*.* The List Sorting fully-corrected *t*-score was used as a primary explanatory variable, representing a behavioral marker of working memory.

#### Cash choice task

Delayed discounting behavior was assessed at baseline with the single item Cash Choice Task (Wulfert et al., 2002). This task was developed and tested with adolescents aged 11–18 years. A research assistant said to participants: “Let’s pretend a kind person wanted to give you some money. Would you rather have $75 in three days or $115 in 3 months?” and recorded responses. Participants were not included if they responded with “can’t decide.”

### fMRI tasks

#### Emotional n-Back (EN-back) task

The EN-back task has been used to elicit neural activation to working memory (Cohen et al., 2016, Owen et al., 2005). The version used by ABCD was a modified version of the Human Connectome Project (HCP; http://www.humanconnectome.org/; Barch et al., 2013) task, which included happy, fearful, and neutral facial expressions and place stimuli (as a non-emotional, non-social control). Facial stimuli were drawn from the NimStim facial expressions stimulus set (Tottenham et al., 2009) and the Racially Diverse Affective Expressions (RADIATE) stimulus set (Conley et al., 2018); place stimuli were drawn from visual perception studies (O’Craven & Kanwisher, 2000; Park and Chun, 2009). In prior studies, contrasting high (2-back) vs. low (0-back) load conditions evoked neural activation to cognitive load reliably across participants and time (Caceres et al., 2009, Drobyshevsky et al., 2006).

During the EN-back task, participants indicated with a button press whether the stimulus they were viewing was a “Match” or “No Match” to the stimulus target presented at the start of the run (0-back) or two trials back (2-back). A 500 ms fixation preceded each block to alert the participant of a switch in the task condition. Behavioral data were collected to examine accuracy for each of the load conditions (i.e., 0- and 2-back). Each participant included in fMRI analyses completed two runs of the EN-back task that passed ABCD quality control procedures (Hagler et al., 2019). Each run contained four high and four low load blocks (25 s each), and four fixation blocks (15 s each); each high and low load block contained 10 trials (2.5 s each; 2 targets, 2–3 non-target lures and non-lures [i.e., stimuli only presented once]). Across both runs, there were a total of 80 trials for each load condition (8 blocks each), and 20 trials for each stimulus type within each load condition.

#### Monetary incentive delay (MID)

The MID task was used to isolate neural activation related to reward processing, including anticipation and feedback on timed responses that could win or avoid losing money (Knutson et al., 2001). Response durations were set based on a practice session before scanning and adjusted throughout the task based on ongoing task performance so that each participant achieved approximately 60% accuracy. At the beginning of each trial, a cue indicated whether that trial would have an incentive (i.e., reward, loss, neutral) of a large ($5) or small ($0.20) amount. Anticipatory neural activation was collected during a jittered anticipation event (1500–4000 ms) immediately following the incentive cue. Once a target appeared on screen (150–500 ms), the participant pressed a button to either win or avoid losing money in the win and loss conditions, respectively. A feedback message informed the participant if they won or lost money during that trial; each MID run was 5-minutes, 42 s.

Each participant included in fMRI analyses completed two runs of the MID task that passed ABCD quality control procedures (Hagler et al., 2019). Each run contained 20 reward, 20 loss, and 10 neutral anticipation trials. The adaptive algorithm aimed for each participant to have 24 positive and 16 negative feedback trials for reward and loss conditions. Across both runs, there were a total of 40 trials for each valenced anticipation condition (i.e., reward, loss), 20 neutral anticipation trials, and an average of 48 positive and 32 negative feedback trials.

### Imaging acquisition and processing

#### Data acquisition

Casey and colleagues (2018) described the full ABCD imaging protocol and data acquisition procedures. Participants were scanned across 21 sites within the United States using multiband echo planar imaging and either a 3 Tesla Siemens Prisma, General Electric 750, or Philips scanner; Casey et al. (2018) reported the full scan parameters used to ensure compatibility across scanners. Participants were asked to complete a 90–120 min scan protocol including T1- and T2-weighted structural images, two resting-state functional images, a diffusion weighted image, and six task-dependent functional images (two runs of three tasks with task order randomized across families); 79% of participants completed the full scan protocol over 1–2 sessions no more than a week apart. All participants completed a mock scan with motion discovery prior to scanning.

#### Pre-processing

We downloaded the ABCC fMRI derivative files from the National Institute of Mental Health Data Archive (https://nda.nih.gov/edit_collection.html?id=3165), which was pre-processed using the Developmental Cognition and Neuroimaging (DCAN) Lab’s processing pipeline. DCAN Lab’s ABCD-HCP-Pipeline (https://github.com/DCAN-Labs/abcd-hcp-pipeline) produced pre-processed images with the following steps completed: 1) de-meaned and de-trended with respect to time, 2) general linear model (GLM) used to denoise the data, and included signal from white matter, cerebrospinal fluid, global signal, and movement variables, and 3) band-pass filtered between 0.008 and 0.09 Hz using a 2nd order Butterworth filter. Subsequently, the pipeline also applied global signal regression, respiratory motion filter, and motion censoring. For more details, please visit their webpage (https://nda-abcd-collection-3165.readthedocs.io/latest/derivatives/) and refer to the ABCC publication (Feczko et al., 2021).

DCAN Lab’s ABCD-HCP-Pipeline output processed fMRI data in volume and surface spaces. We used the ABCC fMRI dense timeseries derivative to generate subject-level activation maps using the DCAN Lab’s ABCD fMRI pipeline (Level 1; https://github.com/DCAN-Labs/abcd-bids-tfmri-pipeline). Activation maps were spatially smoothed (FWHM = 4 mm), and the first two volumes of each run were discarded to allow for magnetization equilibration. We only included individual scan series that were recommended for use by the quality control ABCD team (Hagler et al., 2019). We randomly selected 5% of subjects to visually inspect subject-level *z*-statistic significance maps for all three contrasts of interest to ensure that registration was applied accurately.

#### Subject-specific functional regions of interest and neural activation

**Fig. 2:**
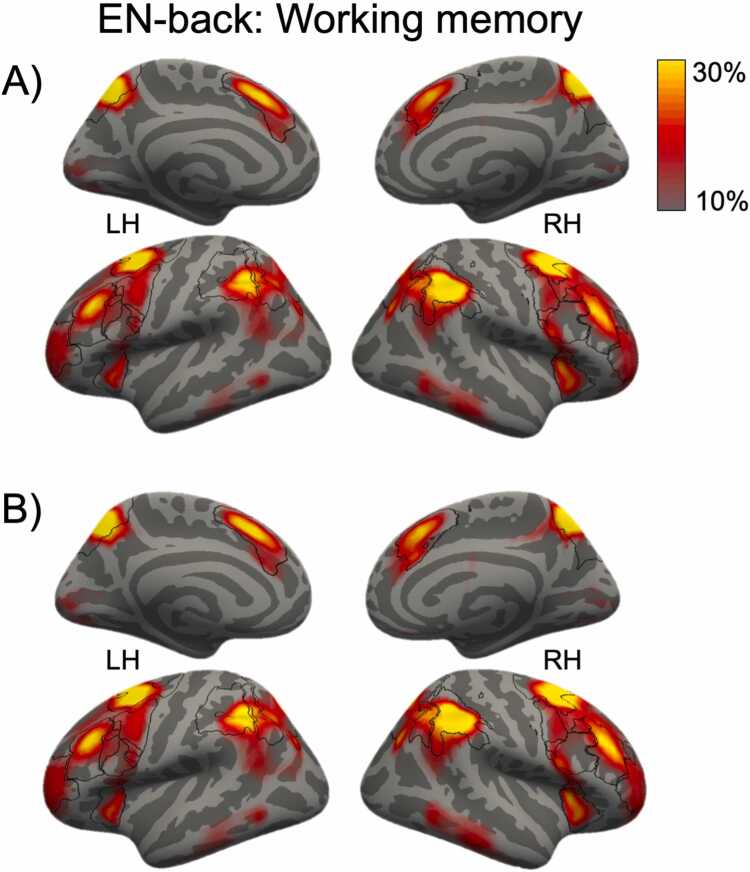
Cortical probabilistic map for fMRI EN-back task. *Note.* Probabilistic map depicting overlapping cortical surface activation during the fMRI emotional n-back (EN-back) task (contrast: 2-back vs. 0-back). Scale reflects the percentage of participants who exhibited significant activation in that voxel (*z* > 2.58). Multiple demand network regions used as search spaces for the subject-specific regions are overlaid with a black outline. Brain images are registered to fsaverage space; LH = left hemisphere; RH = right hemisphere.

**Fig. 3:**
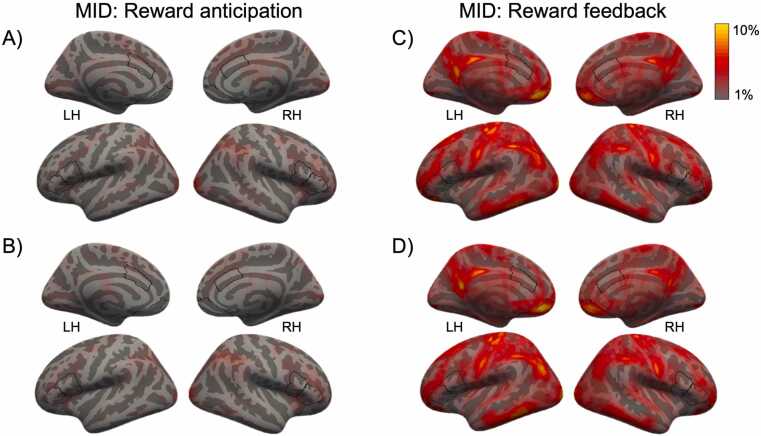
Cortical probabilistic map for fMRI MID task. *Note.* Probabilistic atlases depicting overlapping cortical activation during the fMRI monetary incentive delay (MID) task (contrasts: reward vs. neutral anticipation; positive vs. negative reward feedback [i.e., reward success vs. failure]). Images are shown for the discovery (A/C) and replication (B/D) samples during the reward anticipation (A/B) and feedback periods (C/D). Scale reflects the percentage of participants who exhibited significant activation in that voxel (*z* > 2.58). Cortical reward network regions used as search spaces for the subject-specific regions are overlaid with a black outline. Brain images are registered to fsaverage space; LH = left hemisphere; RH = right hemisphere.

**Fig. 4:**
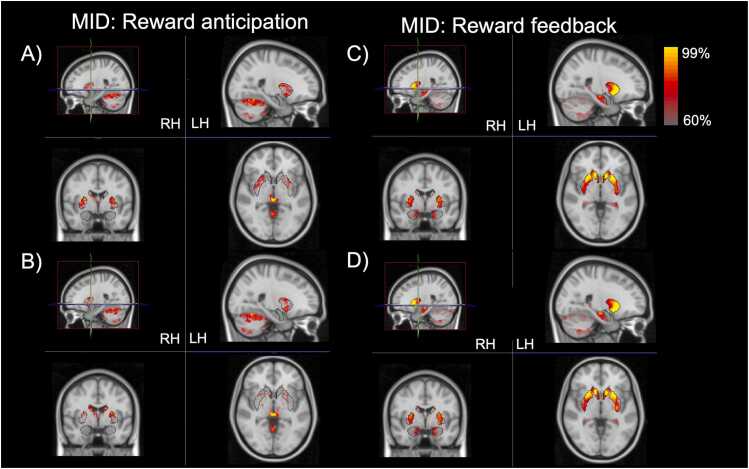
Subcortical probabilistic map for fMRI MID task. *Note.* Probabilistic atlases depicting overlapping subcortical activation during the fMRI monetary incentive delay (MID) task (contrasts: reward vs. neutral anticipation; positive vs. negative reward feedback). Images are shown for the discovery (A/C) and replication (B/D) samples during the reward anticipation (A/B) and feedback periods (C/D). Scale reflects the percent of participants who exhibited significant activation in that voxel (*z* > 2.58). Subcortical volumes are loaded on a Human Connectome Project template brain (MNI152_t1_2mm); MNI coordinates: x = 33, y = 62, z = 35. Subcortical reward regions used as search spaces for the subject-specific regions are overlaid with a black outline. LH = left hemisphere; RH = right hemisphere.

Using the subject-level *z*-statistic significance maps generated by the ABCC first-level GLM processing scripts, we generated subject-specific functional regions of interest (ss-fROIs) for the three neural contrasts of interest. We defined ss-fROIs using an approach similar to the Group-constrained Subject-Specific method (GSS, https://evlab.mit.edu/funcloc/; Fedorenko et al., 2010), by extracting the top 10% of activation within search spaces selected based on prior literature instead of search spaces generated by sample group-constrained activation maps. Regions selective for cognitive control were extracted from 10 bilateral fronto-parietal multiple demand (MD) network parcels defined by Fedorenko and colleagues (2013); these regional search spaces are depicted in Fig. 2 as black outlines. Regions selective for reward processing were extracted from FreeSurfer parcellations (Fischl et al., 2002) that have been reported in publications examining group-level activation during MID (Chaarani et al., 2021, Knutson et al., 2000): bilateral cortical (i.e., caudal anterior cingulate cortex [ACC], pars opercularis and triangularis, and medial-orbitofrontal) and subcortical (i.e., accumbens, caudate, putamen, and thalamus) regions; the cortical regional search spaces are depicted in Fig. 3 as black outlines and subcortical regional search spaces are depicted in Fig. 4 as black outlines.

In addition to the *a priori* reward regions, we performed a data‑driven sensitivity analysis using cortical search spaces defined by overlap of FreeSurfer anatomical parcels with probabilistic activation clusters (excluding visual regions). This procedure resulted in the selection of 11 regions per hemisphere: the isthmus cingulate cortex; the precuneus; and the medial orbitofrontal, postcentral, precentral, rostral middle frontal, superior frontal, caudal middle frontal, lateral orbitofrontal, supramarginal, and inferior parietal gyri. Any FreeSurfer regions that split a single activation cluster were merged to form a single regional search space for ss‑fROI generation, resulting in six search spaces (depicted in Supplemental Figure 1) for the sensitivity analysis.

Custom MATLAB scripts located the voxels within each regional search space, for each individual participant, with the greatest activation (top 10%) to cognitive load (2-back vs. 0-back), reward anticipation (Reward vs. Neutral anticipation), and reward feedback (Positive vs. Negative feedback to a reward cue, i.e., reward success vs. failure) using each participant’s first-level (i.e., subject-level) contrast maps. ss-fROIs were generated for each run of a task, so each participant had two sets of ss-fROIs (run 1 and run 2) for each contrast of interest. Then, neural activation during an independent run was extracted from ss-fROI voxels. For example, ss-fROIs generated from run 1 significance maps were used as the spatial mask when neural activation was extracted from run 2, and vice versa. Neural activation was quantified by contrasting the first-level beta estimates for the relevant contrasts: 1) EN-back high (2-back) minus low (0-back) load represented activation to working memory; 2) MID Reward minus Neutral cue anticipation represented activation to reward anticipation; and 3) MID Positive minus Negative feedback to a reward cue represented activation to reward feedback (i.e., reward success vs. failure). To obtain a single activation value for each contrast, extracted ss-fROI activation was averaged across runs. In the randomly selected 5% of subjects whose subject-level significance maps were inspected, we also visually inspected ss-fROIs for all three contrasts of interest to ensure that registration was applied accurately.

#### Calculating Jaccard coefficients

Within-subject overlap was quantified using the Jaccard coefficient (Jaccard, 1901), which measures the proportional overlap of significant voxels across both runs within each participant (i.e., intersection over union). Subject-level *z*-statistic maps were thresholded at *z* ≥ 2.58 for each run, and binarized maps were generated separately for run 1 and run 2. Jaccard coefficients were then calculated as the number of voxels that were significant in both runs divided by the number of voxels significant in either run. We assessed within-subject overlap on the cortical surface for all three contrasts (EN-back, MID anticipation, and MID feedback), as well as within subcortical volumes for the two MID contrasts.

#### Whole brain correlation analysis

We also conducted exploratory whole brain correlation analyses to identify brain clusters where activation correlated with externalizing behaviors. We averaged *z*-statistic subject-level brain maps within subject across the two runs of each task for the three contrasts of interest. Using FreeSurfer’s mri_glmfit, we computed the correlation between externalizing behaviors and brain activation in the discovery sample; activation maps were spatially smoothed (FWHM = 2 mm) and permutation testing (*n* = 1000) was conducted to correct for multiple comparisons. Only contrasts with surviving clusters after multiple comparison corrections were considered significant and subsequently tested in the replication sample.

#### Resting-state connectivity

We downloaded the tabulated resting-state functional connectivity values from the ABCD Release 5.1. We used the values calculated by the ABCD team (Hagler et al., 2019) reflecting resting-state connectivity between the fronto-parietal network and individual subcortical regions; we used the mean of these values in models examining the relationship between between-network resting-state connectivity and externalizing behavior.

### Analyses

Linear mixed effects models were conducted to test the main and interaction effects of sex, working memory, and reward processing in explaining variance in externalizing behaviors cross-sectionally and at two-year follow-up. These models account for shared variance within families and sites (random intercepts) using RStudio (lmerTest package; Kuznetsova et al., 2017). Table 2 depicts an outline of the general structure of models, which became progressively more complex to allow for stepwise comparisons (described in 2.6-1 below). Model 1 reflects the most parsimonious model, including only relevant covariates. All models included age, the stressful life events composite (i.e., mean of caregiver- and participant-report), and the SES composite (i.e., mean of scaled caregiver-reported parent education and household income) as covariates; we did not include race/ethnicity as a covariate as SES and chronic life stress accounts for much of the racial and ethnic effects related to neural representations of cognitive control and reward processing (Kim et al., 2013; McLaughlin, Weissman, & Bitrán, 2021; Noble et al., 2015; Sheridan et al., 2012). Neural analyses also included framewise displacement during EN-back and MID fMRI tasks (averaged across runs) as a covariate to account for variability explained by in-scanner-motion. Analyses examining externalizing behaviors at two-year follow-up included all baseline covariates as well as baseline externalizing behavior so two-year follow-up estimates could be interpreted as change in externalizing behaviors.

**Table 2:** General model structure for stepwise models.

*Model 1:* Covariates_Age+SES+SLE(+fwd in ‘n’ models)_ → EXT
*Model 2:* Covariates + Sex → EXT
*Model 3*_b_*:* Covariates + Sex + Working Memory_b_ + Reward Processing_b_ → EXT
*Model 4*_b_*:* Covariates + Sex*Working Memory_b_ + Reward Processing_b_ → EXT
*Model 5*_b_*:* Covariates + Sex*Reward Processing_b_ + Working Memory_b_ → EXT
*Model 6*_b_*:* Covariates + Sex*Working Memory_b_ + sex*Reward Processing_b_ → EXT
*Model 7*_b_*:* Covariates + Sex*Working Memory_b_*Reward Processing_b_ → EXT
*Model 3*_n_*:* Covariates + Sex + Working Memory_n_ + Reward Processing_n_ → EXT
*Model 4*_n_*:* Covariates + Sex*Working Memory_n_ + Reward Processing_n_ → EXT
*Model 5*_n_*:* Covariates + Sex*Reward Processing_n_ + Working Memory_n_ → EXT
*Model 6*_n_*:* Covariates + Sex*Working Memory_n_ + sex*Reward Processing_n_ → EXT
*Model 7*_n_*:* Covariates + Sex*Working Memory_n_*Reward Processing_n_ → EXT
*Model 8:* Covariates + Sex*Working Memory_b_*Reward Processing_b_ + Sex*Working Memory_n_*Reward Processing_n_ → EXT

*Note*. Models 3–7 included only behavioral (_b_) or neural (_n_) measures of working memory (List Sorting or fMRI EN-back tasks, respectively) and reward processing (Cash Choice or fMRI MID tasks, respectively; separately for fMRI reward anticipation and feedback contrasts). Model 8 included both behavioral and neural measures. Family and site were included as random intercepts in all models unless otherwise indicated.

Model 2 added the main effect of sex, and Model 3 added the main effects for working memory and reward processing (separately for behavioral and neural measures). Models 4–8 tested a sex by working memory and/or rewarding processing moderation effect (see Table 2 for individual model structures); if sex interaction effects were significant, we conducted post-hoc models separately for boys and girls to probe the interactions. Models 7–8 tested the working memory by reward processing interaction effects. Only Model 8 included both behavioral and neural measures of working memory and reward processing in the same model. Behavioral models included Listing Sorting and Cash Choice tasks as measures of working memory and reward processing, respectively. Neural models included average task activation among MD network ss-fROIs for EN-back and average task activation among reward ss-fROIs for MID (separately for reward anticipation and feedback) as measures of working memory and reward processing, respectively; exploratory analyses were conducted separately for each individual ss-fROI and corrected for multiple comparisons. Unstandardized and standardized coefficients are reported for all models (R effectsize package; Ben-Shachar et al., 2020).

Because the distribution of externalizing *t*-scores had a moderate positive skew (+0.61), we ran exploratory models testing the fit of zero-inflated negative binomial models and negative binomial models, which better account for high positive skews and low base rate behavior (Hilbe, 2011). Due to similar outcomes across models and better estimation of severe externalizing behavior scores among linear models, we only reported linear model outcomes in the main text; fit statistics for the negative binomial models are included in supplemental material and results are available upon request.

#### Model comparisons

To determine which model best explained variance in externalizing behaviors within the discovery sample, we compared models that progressively increased in complexity (Table 2 for general model structure). The preferred model was selected by running stepwise comparisons (ANOVA, *p* < .05) to test whether more complex models significantly outperformed (i.e., explained significantly more variance) more parsimonious models; Akaike Information Criterion (AIC) are reported for each model in supplemental material to convey model fit.

#### Testing replication of preferred model

Once a preferred model was selected, we ran that model with the replication sample using the preferred linear mixed effects model. We also conducted a ridge regression on the discovery sample maintaining only variables that showed a significant effect in the preferred model. We ran 1000 bootstrapped samples of 950 participants (>95% power per bootstrapped sample; procedure also used by Faul et al., 2007) to identify the optimal lambda (i.e., lowest lambda). We then applied that lambda to the replication cohort and compared the *R*^*2*^ value to 1000 permutations of randomly shuffled data. Of note, the ridge regressions do not model random intercepts, so participants within the same family were excluded from ridge regression analyses; within family, the child with the higher externalizing score was retained, and if participants within the same family had equal scores, one child was randomly selected. Site was included in ridge regressions as an additional covariate.

## 3 Results

### Descriptives and attrition

The sample included in analyses largely reflected the general population (i.e., those who do not exhibit significant psychopathology; Table 3). Only 3.49% meet the clinically significant cutoff (*t*-score ≥ 64), while an additional 3.29% scored within the borderline range (*t*-score 60–63), for externalizing behaviors at baseline; thus, approximately 93% of the included sample scored below the clinical and borderline cutoffs for notable externalizing behaviors. Additionally, excluded participants exhibited significantly higher externalizing *t*-scores (*M* = 49.07, *SD* = 11.52) compared to those who were included in the final behavioral (*M* = 44.39, *SD* = 9.50, *t*(5309) = 20.93, *p* < .001) and neural samples (*M* = 43.86, *SD* = 9.25, *t*(6414) = 21.33, *p* < .001). We also compared the two-year follow-up sample to those who were not retained at two-year follow-up (but were included in the baseline sample; *n* = 164); participants with lower baseline SES (*t*(170.96) = -4.7, *p* < .001) and whose caregiver identified them as female (*t*(177.61) = 2.4, *p* = 0.018) were more likely to drop out by two-year follow up.

**Table 3:** Descriptives for psychopathology, working memory, and reward processing variables in discovery & replication samples.

	Baseline			
	Behavioral sample		fMRI sample	
	Discovery	Replication	Discovery	Replication
	*n* = 3714	*n* = 3734	*n* = 1669	*n* = 1596
	*M*(*SD*) or %	*M*(*SD*) or %	*M*(*SD*) or %	*M*(*SD*) or %
CBCL t-score				
Externalizing t-score	44.29(9.35)	44.38(9.55)	43.71(9.28)	43.88(9.24)
Internalizing t-score	47.31(9.90)	47.52(10.06)	47.34(9.68)	47.38(9.98)
List Sorting fully-corrected *t*-score	50.07(9.87)	49.85(9.87)	-	-
Cash Choice Task (% larger, later reward)	59.85	59.64	-	-
EN-back 2- vs. 0-back activation	-	-	1.42(1.52)*	1.45(1.48)*
MID reward anticipation activation	-	-	0.05(0.33)*	0.04(0.32)*
MID reward feedback activation	-	-	0.19(0.40)*	0.19(0.40)*

*Note*. Descriptive statistics for psychopathology, working memory, and reward processing variables of interest. CBCL = Child behavior checklist; EN-back = emotional n-back; MID = monetary incentive delay. Asterisks indicate level of significance (*t*-test, *mu* > 0) for fMRI contrast values averaged across all subject-specific fROIs within network (uncorrected).

* *p* < .001.

### Variability of subject-level significance maps

#### Between-subject variability: working memory

We generated an exploratory probabilistic map of voxels that exhibited significantly greater activation (*z*-statistic > 2.58) during 2-back vs. 0-back contrast of the EN-back fMRI task in the baseline discovery sample (Fig. 2). The maximum number of participants who showed significant activation in the same voxel was 40% of the sample. Despite substantial functional variability, we saw a pattern of activation across several frontal and parietal regions that looked similar to the pattern seen in adults (Fedorenko et al., 2013) and children ages 4–12 years (Schettini et al., 2023).

#### Between-subject variability: reward processing

We included probabilistic atlases for cortical surface (Fig. 3) and subcortical volume (Fig. 4), which depict overlap across subject-level significance maps for both contrasts of interest in the MID task: anticipation of reward vs. neutral cue, positive vs. negative feedback for reward trials. The cortical atlases showed substantial variability across participants; we observed a maximum of 5.21% of participants exhibiting overlap for reward anticipation and a maximum of 10% of participants exhibiting overlap for reward feedback. Notably, the figures depicting the MID atlases use a scale below the minimum threshold depicted in the EN-back probabilistic atlas in Fig. 2; using the same scale produced blank maps for the MID contrasts, highlighting the high variability across participants in the cortical surface MID subject-level significance maps. In contrast, we observed low between-subject variability in the subcortical volume probabilistic atlases; regions such as the caudate, putamen, and nucleus accumbens showed high overlap, with some voxels exhibiting significance among as many as 99% of participants.

#### Within-subject variability: Jaccard Coefficients

**Fig. 5:**
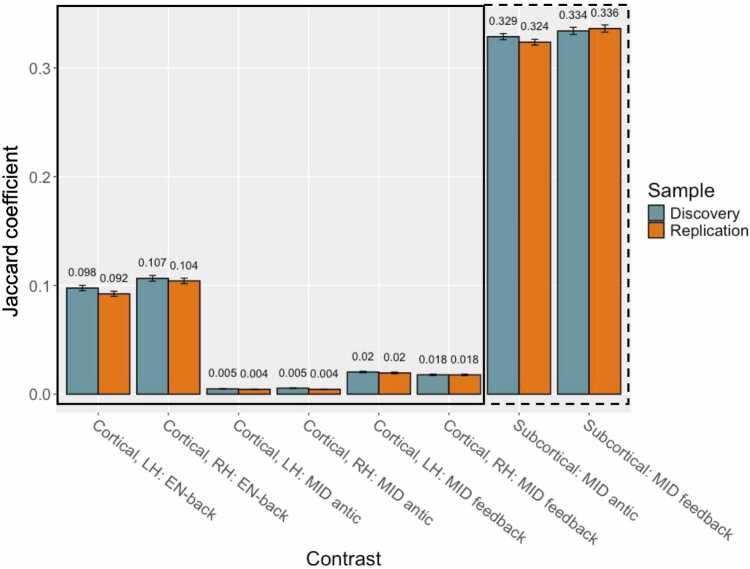
Within-subject overlap for fMRI task activation. *Note*. Jaccard coefficient (i.e., intersection over union) depicting the within-subject overlap between run 1 and run 2 subject-level significance maps for the discovery and replication samples; portrayed separately for cortical (within solid line) and subcortical (within dashed line) tests. Bars and values reflect group mean, and error bars reflect standard error; LH = left hemisphere, RH = right hemisphere, EN-back = working memory contrast, MID antic = MID reward anticipation contrast, MID feedback = MID reward feedback contrast.

Given the high between-subject variability observed in the probabilistic atlases, we also assessed within-subject variability using the Jaccard method (i.e., intersection over union). For the cortical maps, we observed strong positive skews (i.e., large proportion of small Jaccard coefficients) within both the discovery and replication samples. Although the EN-back Jaccard coefficients (means < 0.20) were higher than either of the MID contrasts (means < 0.05; Fig. 5), the EN-back Jaccard coefficients still indicate high within-subject variability across runs. In contrast, the subcortical maps produced a normal distribution of coefficients with larger values (means range 0.32–0.34; Fig. 5), more consistent with what we would expect in fMRI data, highlighting greater within-subject overlap of subcortical neural activation during the MID task.

### Selecting the preferred model using the discovery sample

#### Do behavioral measures explain variability in externalizing behaviors?

Model 6, which included covariates and an interaction between sex and both of the behavioral measures (i.e., List Sorting and Cash Choice task) was selected as the preferred model (Supplemental Table 1). We observed a negative relationship between List Sorting and externalizing behaviors as well as SES and externalizing behaviors; in addition, we found a positive association between stressful life events and externalizing behaviors (Table 4). The sex by List Sorting interaction effect was significant, and in post-hoc analyses, lower List Sorting scores were related to more externalizing behaviors only among boys. In post-hoc models, the SES and stressful life events main effects remained significant in both sexes, but the main effect of Cash Choice task was not significant in either sex. We also observed a negative main effect of age in the model, but when models were run separately by sex (Supplemental Table 2), age was no longer significant for either sex.

**Table 4:** Fixed and random effects for preferred baseline multilevel model: Behavioral.

Fixed Effects	β(*SE*)	CIs	df	*t*-value	β(*SE*)	CIs	df	*t*-value
	Unstandardized				Standardized			
Intercept	58.65(2.69)	[53.4, 63.93]	3357.86	21.8***	0.05(0.04)	[-0.03, 0.13]	91.87	1.19
List Sorting	-0.09(0.02)	[-0.13, −0.04]	3674.00	-4.04***	-0.09(0.02)	[-0.14, −0.05]	3674.00	-4.04***
Sex	-5.55(1.58)	[-8.64, −2.46]	3641.38	-3.52***	-0.08(0.05)	[-0.18, 0.01]	3634.41	-1.68
Cash Choice	0.48(0.42)	[-0.34, 1.30]	3512.65	1.15	0.05(0.04)	[-0.04, 0.14]	3512.65	1.15
Age (months)	-0.05(0.02)	[-0.09, −0.01]	3504.84	-2.69**	-0.04(0.02)	[-0.07, −0.01]	3504.84	-2.69**
Stressful events	0.53(0.09)	[0.35, 0.71]	3581.60	5.71***	0.09(0.02)	[0.06, 0.13]	3581.60	5.71***
SES Composite	-5.67(0.94)	[-7.52, −3.84]	2293.68	-6.04***	-0.11(0.02)	[-0.14, −0.07]	2293.68	-6.04***
List Sorting x Sex	0.10(0.03)	[0.04, 0.15]	3647.23	3.16**	0.10(0.03)	[0.04, 0.16]	3647.23	3.16**
Cash Choice x Sex	-1.19(0.60)	[-2.37, −0.02]	3451.02	-2.00*	-0.13(0.06)	[-0.25, 0.00]	3451.02	-2.00*
	Marginal *R*^*2*^: 0.035							
Random Effects	Variance(*SD*)	Levels			Variance(*SD*)			
Family:Site	33.67(5.8)	3190			0.38(0.62)			
Site	0.88(0.94)	22			0.01(0.1)			
Residual	49.89(7.06)				0.57(0.76)			
	ICC: 0.41							
	Conditional *R*^*2*^: 0.430							

*Note*. Fixed and random effects for preferred multilevel model explaining variance in baseline (*n* = 3714) externalizing *t*-scores using demographics (i.e., age, socioeconomic status (SES), life stressors, sex), behavioral List Sorting and Cash Choice tasks, and random intercepts (family nested within site). Unstandardized and standardized estimates are reported; beta coefficient estimates = β; *SE* = standard error; *SD* = standard deviation. Marginal *R²* reflects the proportion of variance explained by the fixed effects, and conditional *R²* reflects variance explained by both fixed and random effects; ICC = intraclass correlation coefficient. Asterisks indicate level of significance (uncorrected).

* *p* < .05;

** *p* < .01;

*** *p* < .001

#### Do fMRI measures explain variability in externalizing behaviors?

The primary variables of interest tested in the fMRI analyses were sex, EN-back 2-back vs. 0-back, and MID reward (anticipation and feedback conducted separately). Model 2 was selected as the preferred model (see Supplemental Table 3 for fit statistics and model comparisons), which only included sex and covariates (age, SES, life stressors, framewise displacement; Supplemental Table 4). Models including interaction effects between working memory and reward processing neural systems were examined through both linear interaction effects (Supplemental Table 3, Models 7a and 7 f) and functional interactions (i.e., resting-state connectivity; Supplemental Table 3, Models 8a/9a and 8a/9 f). None of the models that included fMRI neural measures outperformed Model 2. Models tested with only neural measures (i.e., task activation and framewise displacement, but no other covariates) still showed null neural effects (*p* > .05) for the brain-behavior relationships. In models that included both behavioral and neural measures, we used the large subset of participants with available behavioral and imaging data at baseline; Model 2, which only included sex and covariates, was also selected as the preferred model (Supplemental Table 5).

To address potential concerns about ROI specification, we conducted a data-driven sensitivity analysis in which ss-fROIs were defined based on overlap with probabilistic activation hotspots rather than *a priori* anatomical boundaries. Results from this approach closely mirrored those of the primary analysis, with no observed associations between reward-related activation and externalizing behavior (see Supplemental Figure 1 for regional overlay on MID probabilistic maps; statistical results available upon request).

#### Predicting externalizing behaviors two-years later using behavioral measures

In the two-year follow-up analyses predicting externalizing behaviors from behavioral baseline predictors, Model 7 was selected as the preferred model (Supplemental Table 6). This model included covariates (baseline externalizing behaviors, age, stressful life events, and SES), sex, and an interaction between baseline List Sorting and Cash Choice tasks (Table 5). In post-hoc analyses, the negative effect of List Sorting was only significant in models including participants who provided the “larger later” response during the Cash Choice task and there was no relationship in models including only participants who provide the “smaller sooner” response (Supplemental Table 7). In models for participants who provided the “larger later” response, there was also a weak interaction effect between sex and List Sorting, and the main effect for more stressful life events at baseline predicting more externalizing behaviors two years later was observed in both Cash Choice response groups when run separately (Supplemental Table 7).

**Table 5:** Fixed and random effects for preferred two-year follow-up multilevel model: Behavioral.

Fixed Effects	β(*SE*)	CIs	df	*t*-value	β(*SE*)	CIs	df	*t*-value
	Unstandardized				Standardized			
Intercept	11.48(2.46)	[6.66, 16.31]	3105.66	4.66***	-0.02(0.02)	[-0.07, 0.02]	177.52	-1.03
Baseline EXT	0.64(0.01)	[0.61, 0.66]	3480.63	50.1***	0.65(0.01)	[0.62, 0.67]	3480.63	50.10***
List Sort	0.01(0.02)	[-0.03, 0.04]	3450.8	0.33	0.01(0.02)	[-0.03, 0.05]	3450.8	0.33
Cash Choice	3.97(1.21)	[1.6, 6.34]	3421.55	3.28**	0.06(0.03)	[0.01, 0.11]	3467.77	2.43*
Sex	-0.19(0.23)	[-0.65, 0.27]	3477.02	-0.8	-0.02(0.03)	[-0.07, 0.03]	3477.02	-0.80
Age (months)	0.01(0.01)	[-0.02, 0.04]	3122.56	0.89	0.01(0.01)	[-0.01, 0.04]	3122.57	0.89
Stressful events	0.23(0.07)	[0.09, 0.37]	3187.47	3.21**	0.04(0.01)	[0.02, 0.07]	3187.48	3.21**
SES composite	1.00(0.71)	[-0.38, 2.39]	1335.48	1.41	0.02(0.01)	[-0.01, 0.04]	1335.51	1.41
List Sorting x CCT	-0.07(0.02)	[-0.11, −0.02]	3432.36	-2.86**	-0.07(0.03)	[-0.12, −0.02]	3432.36	-2.86**
	Marginal *R*^*2*^: 0.430							
Random Effects	Variance(*SD*)	Levels			Variance(*SD*)			
Family:Site	13.5(3.67)	3057			0.16(0.4)			
Site	0.03(0.17)	22			0.00(0.02)			
Residual	33.91(5.82)				0.4(0.64)			
	ICC: 0.29							
	Conditional *R*^*2*^: 0.593							

*Note*. Fixed and random effects for preferred multilevel model predicting two-year follow-up externalizing (EXT) *t*-scores two years later (*n* = 3550) using baseline demographics (age, socioeconomic status (SES), stressful life events, sex), baseline behavioral List Sorting and Cash Choice tasks (CCT), and random intercepts (family nested within site). Unstandardized and standardized estimates are reported; beta coefficient estimates = β; *SE* = standard error; CIs = 95% confidence intervals; *SD* = standard deviation. Marginal *R²* reflects the proportion of variance explained by the fixed effects, and conditional *R²* reflects variance explained by both fixed and random effects; ICC = intraclass correlation coefficient. Asterisks indicate level of significance (uncorrected).

* *p* < .05;

** *p* < .01;

*** *p* < .001

#### Neural measures do not improve prediction of externalizing behaviors 2 years later

The results from the two-year follow-up models for imaging data were similar to the baseline models; adding neural measures of working memory and reward did not significantly improve model performance compared to models that only included covariates (baseline externalizing behaviors, age, stressful life events, and SES, and framewise displacement for each fMRI task). Although Model 7a (included a three-way interaction between sex, EN-back working memory, and MID reward anticipation) explained marginally more variance than Model 6a (only included interactions between sex and each of the fMRI tasks; Supplemental Table 8), Model 7a did not outperform Model 1 (*χ*^*2*^ (7) = 9.08, *p* = .247); therefore, Model 1with only covariates (baseline externalizing, age, stressful life events, SES, and framewise displacement) was selected as the preferred model in the neural sample.

In the two-year follow-up sub-sample, Model 4 (which included covariates, sex, List Sorting, and Cash Choice Task) was selected as the preferred model; even though Model 4 had the highest AIC, stepwise comparisons showed it explained significantly more variance than the three more parsimonious models (see Supplemental Table 9). Model 4 showed that more baseline stressful life events, Cash Choice task “larger later” response, male sex, and lower List Sorting scores were marginally related to more externalizing behaviors two years later (Table 6); the sex main effect was not significant in standardized models.

**Table 6:** Fixed and random effects for preferred two-year follow-up multilevel model: Behavioral and imaging.

Fixed Effects	β(*SE*)	CIs	*df*	*t*-value	β(*SE*)	CIs	*df*	*t*-value
	Unstandardized				Standardized			
Intercept	14.25(3.83)	[6.80, 21.76]	1380.99	3.72***	-0.05(0.04)	[-0.12, 0.03]	140.67	-1.20
Baseline EXT	0.63(0.02)	[0.59, 0.67]	1488.46	32.01***	0.64(0.02)	[0.60, 0.68]	1488.46	32.01***
Sex	-4.49(1.95)	[-8.31, −0.69]	1490.21	-2.31*	0.00(0.04)	[-0.07, 0.08]	1453.17	0.10
List Sorting	-0.07(0.03)	[-0.12, −0.02]	1487.85	-2.54*	-0.07(0.03)	[-0.13, −0.02]	1487.85	-2.54*
Cash Choice	0.71(0.36)	[0.01, 1.42]	1476.09	1.97*	0.08(0. 04)	[0.00, 0.16]	1476.09	1.97*
Age (months)	0.02(0.02)	[-0.02, 0.07]	1416.74	1.09	0.02(0.02)	[-0.02, 0.06]	1416.74	1.09
Stressful events	0.23(0.11)	[0.02, 0.44]	1349.94	2.12*	0.04(0.02)	[0.00, 0.08]	1349.94	2.12*
SES Composite	-0.02(1.19)	[-2.33, 2.32]	898.83	-0.01	0.00(0.02)	[-0.04, 0.04]	898.83	-0.01
EN-back fwd	0.04(0.82)	[-1.55, 1.64]	1490.42	0.05	0.00(0.03)	[-0.05, 0.05]	1490.42	0.05
MID fwd	1.48(1.16)	[-0.80, 3.73]	1488.85	1.28	0.03(0.03)	[-0.02, 0.08]	1488.85	1.28
Sex x List Sorting	0.09(0.04)	[0.02, 0.16]	1489.35	2.37*	0.09(0.04)	[0.02, 0.17]	1489.35	2.37*
	Marginal *R*^*2*^: 0.422							
Random Effects	Variance(*SD*)	Levels			Variance(*SD*)			
Family:Site	10.81(3.29)	1382			0.13(0.36)			
Site	0.09(0.3)	22			0.00(0.03)			
Residual	36.16(6.01)				0.45(0.67)			
	ICC: 0.23							
	Conditional *R*^*2*^: 0.556							

*Note*. Fixed and random effects for preferred multilevel model explaining variance in two-year follow-up externalizing (EXT) *t*-scores using baseline externalizing *t*-score for the sub-sample who have available behavioral and imaging data (*n* = 1504) using demographics (age, socioeconomic status (SES), life stressors, sex), behavioral List Sorting and Cash Choice tasks, and random intercepts (family nested within site). Unstandardized and standardized estimates are reported; degrees of freedom (*df*); beta coefficient estimates = β; *SE* = standard error; *SD* = standard deviation. Marginal *R²* reflects the proportion of variance explained by the fixed effects, and conditional *R²* reflects variance explained by both fixed and random effects; ICC = intraclass correlation coefficient. Asterisks indicate level of significance (uncorrected).

* *p* < .05;

** *p* < .01;

*** *p* < .001

### Replication sample

#### Linear mixed effects model

**Fig. 6:**
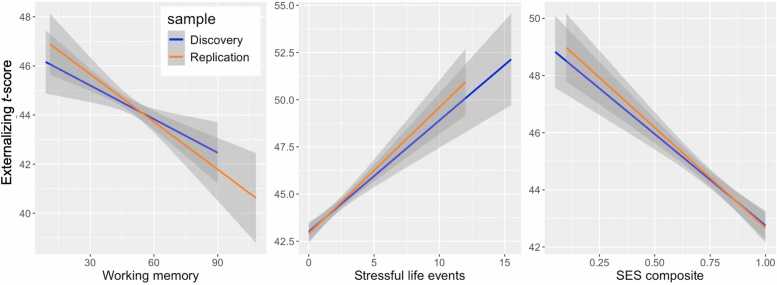
Externalizing correlations with working memory, stressful life events, and SES. *Note*. Linear relationships between the List Sorting task (working memory), stressful life events, and socioeconomic status (SES) with baseline externalizing behaviors in the discovery and replication samples; 95% confidence intervals depicted. All *p* < .001, except the replication working memory which is only *p* < .01.

To assess whether results replicate, we ran the preferred models in the replication sample. In the baseline sample (Table 7), the relationships between lower List Sorting, more stressful life events, and lower SES with more externalizing behaviors also were significant in the replication sample (Fig. 6). In standardized (but not unstandardized) models, boys showed higher caregiver-reported externalizing behaviors than girls; in the discovery sample this effect was only observed in the unstandardized but not standardized models. In contrast to the discovery sample, the interaction effect between sex and List Sorting was not significant, and the negative relationship between List Sorting and externalizing behaviors was significant regardless of sex. In the two-year follow-up analyses, only the main effect of more stressful life events at baseline significantly predicted externalizing behaviors two years later (Fig. 6) after controlling for baseline externalizing behaviors (Table 8); the interaction between List Sorting and CCT was not significant like it was in the discovery sample.

**Table 7:** Preferred multilevel model in the baseline replication sample.

Fixed Effects	β(*SE*)	CIs	df	*t*-value	β(*SE*)	CIs	df	*t*-value
	Unstandardized				Standardized			
Intercept	53.16(2.65)	[47.98, 58.38]	3227.60	20.02***	0.08(0.04)	[0.00, 0.16]	86.35	1.96
List Sorting	-0.06(0.02)	[-0.1, −0.02]	3662.48	-2.76**	-0.06(0.02)	[-0.1, −0.02]	3662.48	-2.76**
Sex	-1.43(1.61)	[-4.57, 1.72]	3652.70	-0.89	-0.15(0.05)	[-0.24, −0.05]	3627.37	-2.92**
Cash Choice	-0.62(0.41)	[-1.42, 0.18]	3295.01	-1.53	-0.07(0.04)	[-0.15, 0.02]	3295.01	-1.53
Age (months)	-0.01(0.02)	[-0.05, 0.03]	3328.12	-0.65	-0.01(0.02)	[-0.04, 0.02]	3328.12	-0.65
Stressful events	0.55(0.09)	[0.37, 0.73]	3644.14	6.03***	0.10(0.02)	[0.07, 0.13]	3644.14	6.03***
SES Composite	-6.38(0.97)	[-8.28, −4.5]	2451.99	-6.60***	-0.12(0.02)	[-0.15, −0.08]	2451.99	-6.60***
Sex x List Sorting	0.00(0.03)	[-0.06, 0.06]	3653.25	0.02	0.00(0.03)	[-0.06, 0.06]	3653.25	0.02
Sex x Cash Choice	0.78(0.61)	[-0.4, 1.97]	3379.12	1.29	0.08(0.06)	[-0.04, 0.21]	3379.12	1.29
	Marginal *R*^*2*^: 0.033							
Random Effects	Variance(*SD*)	Levels			Variance(*SD*)			
Family:Site	38.45(6.20)	3214			0.42(0.65)			
Site	1.07(1.04)	22			0.01(0.11)			
Residual	48.53(6.97)				0.53(0.73)			
	ICC: 0.45							
	Conditional *R*^*2*^: 0.467							

*Note*. Fixed and random effects for preferred multilevel model in the replication sample explaining variance in baseline (*n* = 3734) externalizing *t*-scores using demographics (age, socioeconomic status (SES), life stressors, sex), behavioral List Sorting and Cash Choice tasks, and random intercepts (family nested within site). Unstandardized and standardized estimates are reported; beta coefficient estimates = β; SE = standard error; CIs = 95% confidence intervals; SD = standard deviation. Marginal *R²* reflects the proportion of variance explained by the fixed effects, and conditional *R²* reflects variance explained by both fixed and random effects; ICC = intraclass correlation coefficient. Asterisks indicate level of significance (uncorrected).

** *p* < .01;

*** *p* < .001

**Table 8:** Preferred multilevel model in the two-year follow-up replication sample.

Fixed Effects	β(*SE*)	CIs	df	*t*-value	β(*SE*)	CIs	df	*t*-value
	Unstandardized				Standardized			
Intercept	12.86(2.47)	[8.05, 17.77]	3142.84	5.22***	0.00(0.03)	[-0.06, 0.05]	67.96	-0.09
Baseline EXT	0.61(0.01)	[0.59, 0.64]	3540.42	48.53***	0.63(0.01)	[0.61, 0.66]	3540.42	48.53***
List Sorting	-0.02(0.02)	[-0.06, 0.02]	3538.71	-1.10	-0.02(0.02)	[-0.06, 0.02]	3538.71	-1.10
Cash Choice	-0.66(1.22)	[-3.05, 1.72]	3491.56	-0.54	0.01(0.03)	[-0.04, 0.06]	3442.90	0.55
Sex	-0.02(0.24)	[-0.48, 0.44]	3519.58	-0.09	0.00(0.03)	[-0.05, 0.05]	3519.58	-0.09
Age (months)	0.03(0.01)	[0.00, 0.05]	3344.93	1.77	0.02(0.01)	[0.00, 0.05]	3344.93	1.77
Stressful events	0.32(0.07)	[0.18, 0.46]	3390.42	4.55***	0.06(0.01)	[0.03, 0.09]	3390.42	4.55***
SES Composite	0.07(0.74)	[-1.38, 1.52]	1877.52	0.10	0.00(0.01)	[-0.03, 0.03]	1877.52	0.10
List Sorting x CCT	0.02(0.02)	[-0.03, 0.06]	3496.67	0.66	0.02(0.03)	[-0.03, 0.07]	3496.67	0.66
	Marginal *R*^*2*^: 0.418							
Random Effects	Variance(*SD*)	Levels			Variance(*SD*)			
Family:Site	14.47(3.8)	3075			0.17(0.42)			
Site	0.35(0.59)	22			0.00(0.06)			
Residual	33.75(5.81)				0.40(0.63)			
	ICC: 0.31							
	Conditional *R*^*2*^: 0.595							

*Note*. Fixed and random effects for preferred multilevel model in the replication sample explaining variance in two-year follow-up (*n* = 3577) externalizing (EXT) *t*-scores using demographics (age, socioeconomic status (SES), life stressors, sex), behavioral List Sorting and Cash Choice (CCT) tasks, and random intercepts (family nested within site). Unstandardized and standardized estimates are reported; beta coefficient estimates = β; *SE* = standard error; *SD* = standard deviation. Marginal *R²* reflects the proportion of variance explained by the fixed effects, and conditional *R²* reflects variance explained by both fixed and random effects; ICC = intraclass correlation coefficient. Asterisks indicate level of significance (uncorrected).

** *p* < .01;

*** *p* < .001

**Fig. 7:**
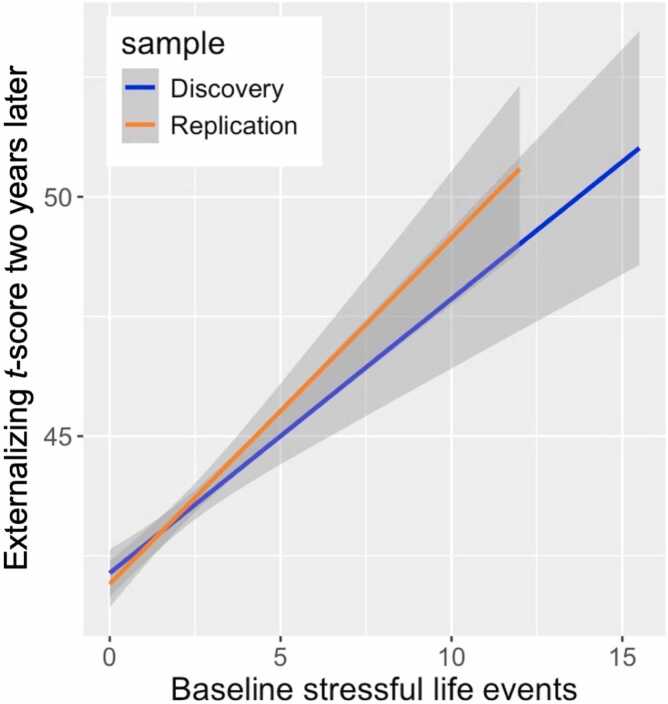
Correlations between stressful life events and externalizing behaviors two-years later. *Note*. Linear relationship between baseline stressful life events with two-year follow-up externalizing behaviors two years later; 95% confidence intervals depicted. *p* < .001 for both discovery and replication models.

We also conducted follow-up analyses to test how robust these findings were when adding additional covariates in the discovery and replication samples. Main effects for SES and stressful life events remained significant in both the discovery and replication samples at baseline when a general cognitive ability composite score (mean of the NIH Toolbox Cognition Battery, fully corrected *t*-scores, excluding List Sorting: Dimensional Change Card Sort, Flanker, Picture Sequence, Pattern Comparison, Picture Vocabulary, and Oral Reading Tests) was added to the model (Supplemental Table 10); List Sorting was trending in the discovery sample and marginally significant in the replication sample. Main effects for List Sorting, SES, and stressful life events remained significant in both the discovery and replication samples at baseline when the CBCL internalizing behavior *t*-score (which includes anxiety, depression. and social withdrawal subscales) was added to the model (Supplemental Table 11). Stressful life events also remained significant in both the two-year follow-up discovery and replication samples after controlling for internalizing behaviors (Supplemental Table 12).

When examining whether statistical assumptions were violated, the linear models demonstrated bimodal residuals and heteroscedasticity. Negative binomial and zero-inflated negative binomial models were tested in the discovery sample (Supplemental Table 13; Supplemental Table 14). Despite different modeling approaches, each showed similar outcomes and visual inspection of normal Q-Q plots (Supplemental Figure 2) revealed that the linear models better explained variability in the higher externalizing scores. Given that predicting clinical levels of externalizing scores (i.e., higher scores) was the primary goal of this study, only linear models are discussed, and negative binomial tables are available upon request.

#### Ridge Regression and Permutation Testing

We conducted bootstrapped 5-fold cross-validation ridge regressions on 1000 samples from the discovery set (Supplemental Table 15) to determine the optimal lambda to be applied to the replication set; because there was a positive skew (+1.57) of the bootstrapped lambda distribution, the median value was selected as the optimal lambda that was applied to the replication set. Significance of the ridge regression applied to the replication sample was assessed by comparing the observed deviance ratio in the baseline sample (*R*^*2*^ = 0.037) and two-year follow-up sample (*R*^*2*^ = 0.426) to a null distribution of deviance ratios generated from 1000 random permutations (Pho et al., 2024) of the baseline or two-year follow-up dataset, respectively. None of the random permutations had a deviance ratio greater than the observed deviance ratio in either baseline or two-year follow-up sample, suggesting that our model performs significantly better than random.

### Exploratory analyses

#### Sensation seeking and positive urgency

Given power limitations of the initially proposed dichotomous measure of reward reactivity (i.e., Cash Choice task responses), we also tested the relationship of two subscales of the UPPS-P Impulsive Behavior Scale (Whiteside and Lynam, 2001). Sensation seeking (i.e., motivation to pursue exciting or pleasurable experiences) and positive urgency (i.e., responses to high-arousal positive emotions) were selected in effort to parallel the MID neural contrasts reward anticipation and reward feedback, respectively. In the discovery sample, both sensation seeking and positive urgency at baseline were strongly related to baseline externalizing behaviors (*p* < .001; tables available upon request); however, only the positive urgency effect was significantly related to externalizing behaviors in the replication sample. In two-year follow-up analyses, sensation seeking was marginally related to externalizing behaviors two years later (*p* < .05; tables available upon request) in both the discovery and replication samples; positive urgency was not significantly associated with externalizing behaviors in either sample.

#### Subject-Specific fROIs: individual region models

**Fig. 8:**
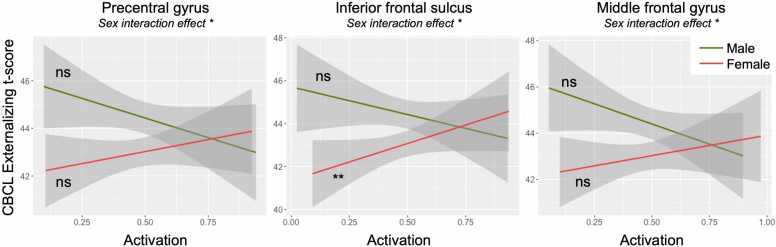
Sex interactions among individual multiple demand network regions during the EN-back task. *Note*. Linear relationships between baseline externalizing behaviors in the discovery sample with activation of individual regions in the multiple demand network during the EN-back task; 95% confidence intervals depicted. Images depict the significance value of the sex interaction effect and main effects of activation on externalizing behaviors when models were conducted separately by sex. * indicates *p* < .05; ** indicates *p* < .01; ns indicates *p* > .05.

Because the hypothesized network activations were not significantly related to externalizing behaviors, we tested whether activation of individual regions (averaged across hemispheres) were related to externalizing behaviors. In the discovery sample, three frontal regions (precentral gyrus, inferior frontal sulcus, middle frontal gyrus; Fig. 8) showed a significant interaction between EN-back working memory activation and sex (*p* < .05, uncorrected), while controlling for age, socioeconomic status, and life stressors; significant effects disappeared when covariates were removed from the model and no interaction effects survived multiple comparison correction. Among models conducted separately by sex in the discovery sample, only one main effect was significant (*p* < .01) in female participants: greater activation of the inferior frontal sulcus correlated with more severe externalizing behaviors. Notably, none of these effects replicated in the replication sample. No interaction or main effects of MID fMRI activation were identified for the reward anticipation or feedback contrasts (*p*’s > .05).

#### Whole brain correlation analysis

**Fig. 9:**
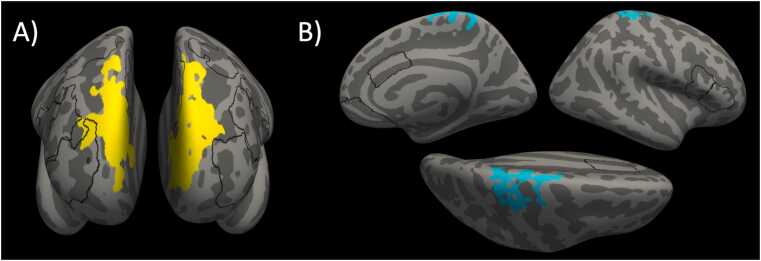
Group-level generalized linear models: Correlation analyses. *Note*. Brain clusters that survived multiple comparison correction in the discovery sample for correlation between baseline externalizing behaviors and activation during the A) EN-back working memory and B) MID reward feedback fMRI contrasts; none of these regions replicated in the replication sample. Black outlines indicate the *a priori* functional regions of interest used to extract ss-fROIs of the A) multiple demand network and B) cortical reward regions, illustrating how the whole brain analyses may have revealed additional relationships not captured by ss-fROI analyses.

We conducted exploratory GLMs to investigate whether any clusters across the whole brain correlated with externalizing behaviors at baseline. In the cortical surface significance maps for working memory, one bilateral brain cluster survived multiple comparison correction in both the left and right hemisphere within the discovery sample (Fig. 9A). In the cortical surface reward feedback contrast of the MID task, one cluster in the right hemisphere survived multiple comparison correction within the discovery sample (Fig. 9B). However, none of these clusters replicated, nor were any additional clusters identified, when exploratory GLMs were conducted within the replication sample. Across the cortical reward anticipation and subcortical reward anticipation and feedback significance maps, no clusters exhibited significant associations between activation and externalizing behaviors in the discovery sample.

## 4 Discussion

In this study, we explored whether behavioral and neural measures of working memory and reward reactivity explained variability in the severity of concurrent externalizing behaviors at ages 9–10 and whether they were related to severity two years later. There were stable relationships between poor working memory (assessed behaviorally), more stressful life events, and lower socioeconomic status (SES) with concurrent externalizing behaviors. After controlling for baseline severity, only stressful life events at baseline significantly explained variability in externalizing behaviors two years later. Contrary to our hypotheses, neither reward reactivity (behavioral or neural) nor neural measures of working memory were related to externalizing behaviors concurrently or at the two-year follow-up for the examined contrasts in this sample. We also did not observe a stable interaction effect between behavioral or neural measures of working memory and reward processing.

Our findings were consistent with the well-documented relationship between SES and life stressors with antisocial behaviors (e.g., Piotrowska et al., 2015; Tolan, 1988). Lower SES is associated with greater exposure to chronic stressors (e.g., neighborhood violence, systemic discrimination, delinquent peer affiliations, food and housing instability, family conflict, and harsh/inconsistent discipline; Baum et al., 1999; Beaver et al., 2008; Moffitt and Caspi, 2001; Scarpa and Ollendick, 2003; Steptoe and Feldman, 2001). Chronic life stressors likely influence externalizing behaviors through both biological and environmental mechanisms. For example, it is possible that stress can impact biological reactivity through allostatic load altering hypothalamic–pituitary–adrenal axis and metabolic responses over time (McEwen, 2017), and may differentially impact children based on their existing biological predispositions (Fagan et al., 2017, Tuvblad et al., 2006). Aside from individual stress, there are other potential explanations for the association between SES and externalizing behaviors, such as intergenerational stress. Parents of children experiencing stressful life events are often also experiencing chronic stressors, which increases risk of childhood externalizing problems, such as conduct problems, oppositional behaviors, and substance use (Fong et al., 2019, Kersey et al., 2024, Kochanova et al., 2021, Thornberry et al., 2009), potentially through exposure to caregiver externalizing behaviors (e.g., interpersonal violence, substance use; Bozzay et al., 2020; Savell et al., 2022). Additionally, lower parental education and income increases the likelihood of working long hours in low-wage jobs, limiting time for direct supervision and increased reliance on older siblings or neighborhood networks for childcare (reviewed by McLoyd, 1998). Given prior research linking lower parental supervision to childhood conduct problems (reviewed by Racz and McMahon, 2011), this may partially account for the observed SES effects. Future research could benefit from further exploration into the mechanisms driving the effect of SES and stressful life events on externalizing behaviors.

We also believe it is important to highlight the relationship between general cognitive control and SES, given that both working memory and SES showed strong negative relationships with externalizing behaviors. Our findings indicate that working memory contributes uniquely to explaining variability in externalizing behaviors beyond the effects of SES and life stressors, supporting theories that impulsive behaviors may arise from poor top-down regulation of emotional and behavioral urges (Beauchaine and McNulty, 2013). Although neural evidence of this effect was not found in this study, our behavioral models suggest that lower working memory is a risk factor for externalizing psychopathology. Notably, fixed effects explained only about 3.5% of variance, suggesting that despite strong statistical significance, there may be limited clinical or practical significance of these findings.

Some important demographic patterns also emerged that are worth discussing. Age effects in this study were only marginally significant in the discovery sample and were not significant in the replication sample, potentially due to the narrow age range (9–10 years), which limited our ability to detect broader developmental trends. Therefore, these data do not preclude the possibility that age effects exist across a wider age range, and future research with more inclusive age ranges may clarify the developmental trajectory of externalizing behaviors.

Sex differences were also observed in both the discovery and replication samples, though we observed inconsistencies in significance across standardized and unstandardized models. This indicates that the sex effect is sensitive to sample characteristics and/or scaling, and may not be a stable relationship. This finding was surprising given the robust body of literature showing that males exhibit higher levels of externalizing behaviors (e.g., aggression, impulsivity, rule-breaking; Loeber and Hay, 1997; Moffitt and Caspi, 2001; Zahn-Waxler et al., 2008). It is possible that externalizing behaviors among females may have been overlooked in previous studies due to differences in symptom expression and use of assessment tools typically normed primarily with male samples (Hartung and Widiger, 1998, Martel, 2013), which may exaggerate the size of sex effects previously reported. The fragile sex effects reported in this study may be explained by our use of CBCL fully corrected *t*-scores, which are normed separately for boys and girls to reduce potential measurement bias. Additionally, sex effects may be more prominent in clinical samples and, given that our sample is primarily below thresholds for clinically relevant externalizing behaviors, such effects were not reliably observed here. Researchers should further investigate how sample and measurement variations influence sex-effects on externalizing behaviors.

Although interactions between sex and working memory, as well as between sex and Cash Choice task response, marginally improved model performance in the discovery sample, these effects did not replicate. In the replication sample, working memory showed a strong main effect regardless of sex, and no effects of Cash Choice task were observed, suggesting the initial interactions were likely sample-specific. Similarly, the observed interaction between working memory and Cash Choice task predicting externalizing behaviors two years later in the discovery sample did not replicate, aligning with prior research indicating that Cash Choice behaviors are largely explained by shared environmental risk factors (Sparks et al., 2014), which were appropriately accounted for in this study.

We further tested the robustness of our primary findings (i.e., SES, behavioral working memory, and stressful life events associated with greater externalizing severity) with several post-hoc analyses. The relationship between working memory and externalizing behaviors was weaker after accounting for a general cognition composite, suggesting that working memory captures relevant variability related to general cognitive effects; if time constraints exist, reducing assessments to just working memory could increase accessibility of cognitive testing for children being assessed for externalizing pathology. Additional models including internalizing behaviors, to account for general psychopathology risk, revealed that while internalizing behaviors were strongly related to baseline externalizing behaviors, our primary findings remained significant. In two-year follow-up models, baseline stressful life events were still significantly associated with externalizing behaviors two years later, whereas baseline internalizing behaviors were not, suggesting that development of externalizing disorders is more closely related to stressful life events than to general psychopathological risk.

It is also important to discuss the null findings of brain-behavior relationships. We observed substantial within-subject variability, indicating that cortical task activation for these contrasts exhibit limited stability and increased noise in this sample. Although the working memory probabilistic maps showed a consistent pattern with prior literature (Fedorenko et al., 2013, Schettini et al., 2023), they also show that at most, only about 30% of participants shared significant activation in any voxel. It is possible that the emotional component of the EN-back task may have negatively impacted reliability, even though the working memory component was the same for both emotional and non-emotional stimuli in the EN-back task; this would be consistent with prior literature that showed lower internal consistency among emotional tasks vs. purely cognitive tasks (reviewed by Elliot et al., 2020), especially in frontal regions (Hassel et al., 2020). The cortical maps for reward reactivity (anticipation and feedback) showed substantial variability between- and within-subjects, suggesting that the MID contrasts used in this study did not reliably capture cortical reward processing in this sample. Additionally, while the subcortical maps showed high consistency between- and within-subjects, we still did not observe significant relationships between subcortical activation and externalizing behavior. One possibility is that despite high coherence among participants in spatial extent of activation, the range of subcortical reward activation may have been too restricted to effectively explain variance in behavior.

Given the limited between-participant overlap observed in the *a priori* cortical regions examined here, it may be unsurprising that robust relationships with externalizing behavior were not detected. Consistent with this interpretation, exploratory whole-brain analyses did not identify additional clusters that replicated across samples, and data‑driven ss‑fROI sensitivity analyses similarly yielded null results. Together, these findings provide preliminary evidence that activation to the MID anticipation and feedback contrasts is not robustly associated with CBCL externalizing scores in this age group. Another potential explanation for the null findings is the low base rate of externalizing behaviors in this sample, which may limit sensitivity to detect brain-behavior associations. Such relationships may be more readily detectable in clinical samples characterized by higher levels of symptom severity. These results motivate future research aimed at clarifying how methodological choices, developmental stage, and symptom severity shape observed brain-behavior relationships.

Future research would likely benefit from examining additional tasks, in addition to other MID and EN-back contrasts, that leverage emotional or motivational stimuli within the context of cognitive tasks to tap into concurrent function of both neural systems at once; some ways to do this would be examining the emotional component of the EN-back task, differentiating large vs. small reward within the MID task, or the HCP’s Conditioned Approach Response Inhibition Task (https://www.humanconnectome.org/study/hcp-lifespan-development/project-protocol/task-protocols-hcp-development). These tasks provide the opportunity to reveal state-dependent functional interactions that may better explain variability in externalizing behaviors. This would be consistent with findings recently reported by Assari and Donovan (2025) regarding the relationship between delinquent behaviors and neural reactivity to emotional stimuli in the ABCD sample. It is also possible that using different neural measures (e.g., other resting state measures, brain structure), polyneuro risk scores, or multi-model neural composites (e.g., Kliamovich et al., 2025; Royer et al., 2024; Xu et al., 2024) may reveal stronger relationships with externalizing behaviors. Because the interaction between biology and environmental risks over time likely maintains and exacerbates externalizing behaviors (Dash et al., 2023, Hicks et al., 2009), it may be difficult to disentangle effects of SES and life stressors from biological effects on behavior throughout the lifespan.

To address concerns about variability in functional organization, especially in cortical regions, we used subject-specific fROIs in our linear models. However, even when using this approach, the neural representations of working memory and reward that we chose to examine in this study were unrelated to baseline and two-year follow-up externalizing behaviors assessed. We also examined whether activation within individual regions or even voxelwise across the whole brain elicited any significant correlations with externalizing behaviors. Despite observing some frontal regions with marginally significant relationships between working memory activation and externalizing behavior in the discovery sample, none of these replicated in the replication sample. Given the observed variability across- and within-subject, it is unsurprising that these brain-behavior relationships, even when using standard GLM analyses, did not replicate across samples. Other researchers have discussed concerns about reliability of the task fMRI measures within the ABCD study (Kennedy et al., 2022) and the impacts of reliability on accuracy of brain-behavior predictive models (Gell et al., 2024). However, it is possible that finer-grain functional connectivity specifically between ss-fROIs, either during task or rest, or perhaps different neural measures (e.g. multi-model neural composites; Kliamovich et al., 2025; Royer et al., 2024; Xu et al., 2024) may reveal neural associations with externalizing behaviors and perhaps show interactions with behavioral task performance or environmental experiences.

Strengths of this study included its multi-method approach and rigorous control for covariates. Analyzing both behavioral and neural measures of working memory and reward processing in the same models provided a comprehensive test of theorized biological vulnerabilities and environmental risk factors of externalizing behaviors (Beauchaine and McNulty, 2013). Using neural measures of reward processing and working memory, in tandem with environmental risk factors, allowed for a more thorough exploration of theorized biological mechanisms underlying externalizing behaviors. Additionally, the key findings, i.e., poor behavioral working memory, low SES, and high stressful life events, were robust even when adding additional control variables to the model, strengthening confidence in the identified relationships with externalizing behaviors. Lastly, we reported novel probabilistic maps depicting substantial between-subject variability observed in the ABCD baseline data, as well as reported substantial within-subject cortical variability among the ABCD fMRI tasks.

Despite these strengths, several limitations should be noted. Children with clinical levels of externalizing behaviors were more likely to be excluded, or have missing data, potentially limiting the generalizability of findings to high-risk populations. Future studies could address this by oversampling children with clinically relevant behaviors or implementing data collection strategies to minimize missing data for children with clinical levels of externalizing behaviors. Additionally, the ABCD sample is highly educated compared to the national average. If the sample is not representative of SES overall, then this limits generalizability of model outcomes to the general population. Lower SES participants were also less likely to return for follow-up assessments and be included within the neuroimaging sub-sample; this pattern is consistent with previously reported greater participation and retention for higher SES individuals in neuroimaging studies (Harnett et al., 2024), which may reflect the increased likelihood of residential instability among low-SES families (Leventhal and Brooks-Gunn, 2000) making longitudinal retention more challenging. Given the strong SES-externalizing behavior relationship at baseline, reduced SES variability in the two-year follow-up sample may have limited our ability to detect SES effects two years later.

The neuroimaging sub-sample also exhibits overrepresentation of white participants compared to Black participants. Because historical and systemic racism has led to persistent racial disparities in SES and stressful life events through mechanisms such as structural racism, hiring discrimination, and educational inequities (Bertrand and Mullainathan, 2004; Campbell & Kaufman, 2006; Reardon et al., 2019), it is unsurprising that racial attrition patterns parallel the SES attrition patterns in this study. Notably, because we controlled for SES and stressful life events in all models, we believe that the social inequities captured by the social constructs of race/ethnicity were adequately accounted for in this study. Researchers have discussed the need for more diverse, representative samples in order to improve generalizability of brain-behavior relationships in large-scale consortia studies, such as ABCD (Marek and Laumann, 2025; Nakua et al., 2024; Xu et al., 2024). Differential retention based on SES and race highlight broader systemic inequities in research participation, emphasizing the need for intentional efforts to address disparities in neuroimaging research to ensure generalizability.

There are also some notable statistical and measurement limitations that are worth discussing. The dichotomous nature of the Cash Choice task, the primary behavioral measure of reward processing, may have limited power to detect a relationship between reward and externalizing behaviors. Additionally, we expected, but did not observe, a fan-shaped interaction effect between working memory and reward reactivity; this may be unsurprising given that fan-shaped interaction effects are particularly difficult to detect (McClelland and Judd, 1993). Detecting the three-way interaction between sex, working memory, and reward reactivity was also constrained by the dichotomous nature of two key behavioral variables (i.e., sex and Cash Choice task), which may further explain the null findings. Caregiver-reported CBCL measures likely also introduce noise compared to objective measures of externalizing behavior (Eisenberg et al., 2019), providing another potential explanation for null findings. Future studies would likely benefit from using continuous behavioral measures and behavioral task measures of externalizing behavior to reduce noise and improve power for complex interaction testing.

Statistical assumptions must also be considered when interpreting results. Studies examining low-base rate behaviors, such as those investigated in this sample, often violate traditional linear model assumptions, particularly normality. This is a well-documented challenge in fMRI research, as fMRI data do not always conform to GLM assumptions (Monti, 2011, Poline and Brett, 2012). However, the central limit theorem suggests that normality violations become less concerning in large samples (Barri, 2019), reducing concerns about misinterpretation in our dataset. Nevertheless, it is important to acknowledge these statistical violations when interpreting findings, particularly in studies including smaller samples.

In conclusion, this study provided evidence that lower working memory (via neuropsychological assessment), lower SES, and more stressful life events were associated with more severe concurrent externalizing behaviors in children, and stressful life events were strongly correlated with increases in externalizing behaviors over two years. These findings contribute to our understanding of the biological and environmental factors influencing externalizing behaviors in children and highlight the need for continued research into neural mechanisms underlying externalizing behavior. Future studies should explore alternative reward processing measures, particularly in high-risk samples, to further clarify their role in externalizing behaviors. Identifying predictors of externalizing behaviors is essential for informing individual- and system-level interventions that could help to mitigate development of severe aggressive and rule-breaking behaviors across the lifespan.

## CRediT authorship contribution statement

**Jennifer Cheavens:** Writing – review & editing, Supervision, Methodology, Conceptualization. **Elana Schettini:** Writing – original draft, Visualization, Methodology, Funding acquisition, Formal analysis, Data curation, Conceptualization. **Zeynep M. Saygin:** Writing – review & editing, Supervision, Methodology, Funding acquisition, Conceptualization.

## Funding

E.S. was supported by the Presidential Fellowship at The Ohio State University and is currently supported by the Department of Veterans Affairs New England MIRECC at VA Connecticut Health Care System. Z.M.S. was supported by the Alfred P. Sloan Foundation, Ohio State University's College of Arts & Sciences, and the Chronic Brain Injury initiative at Ohio State University.

## Declaration of Competing Interest

The authors declare that they have no known competing financial interests or personal relationships that could have appeared to influence the work reported in this paper.

## Data Availability

The authors do not have permission to share data.
