# Supplementary material for: Effects of working memory and reward reactivity on externalizing behaviors in the ABCD study
Source: Dev Cogn Neurosci. 2026 May 25;80:101743. doi: 10.1016/j.dcn.2026.101743 (PMC13265658; doi:10.1016/j.dcn.2026.101743)
Supplement: Supplementary file 1 — Supplementary material [file mmc1.docx]

# Supplemental Materials

## Supplemental Figure 1

Regional search spaces used to generate the data-driven cortical subject-specific functional regions of interest for the monetary incentive delay task sensitivity analysis.


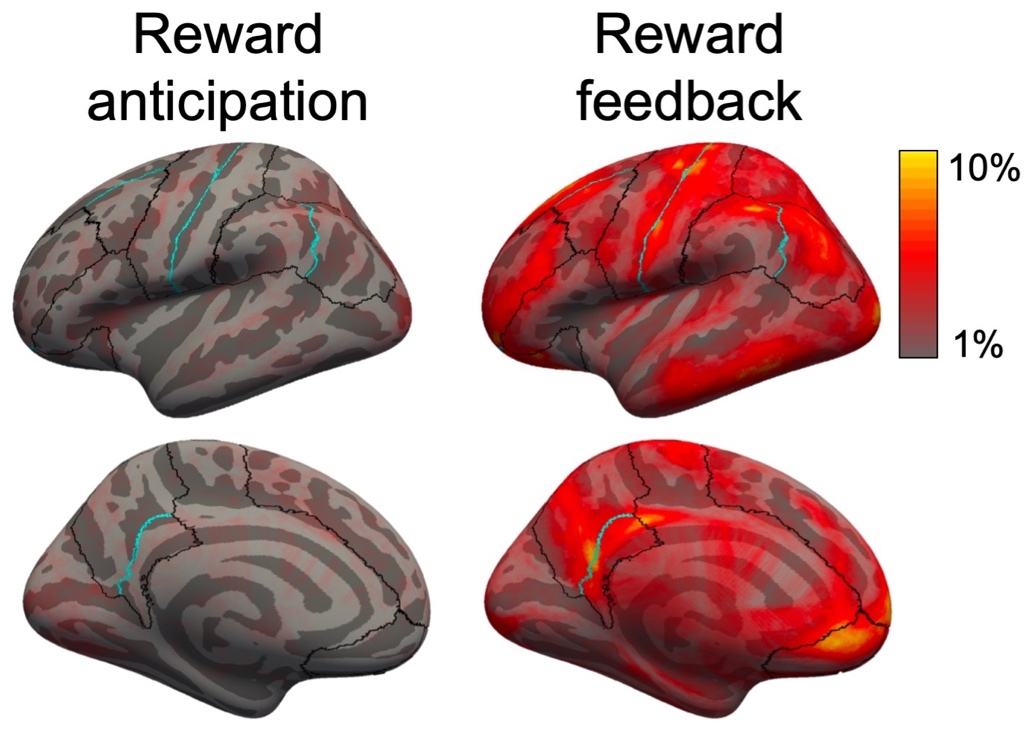


*Note.* Data-driven cortical search spaces overlaid with a black outline on the probabilistic atlases depicting overlapping cortical activation during the fMRI MID task (contrasts: reward vs. neutral anticipation; positive vs. negative reward feedback [i.e., reward success vs. failure]); probabilistic atlases are the same as depicted in Figure 3 but with different regional overlays. Neon blue boundaries indicate boundaries of FreeSurfer regions that were merged. Scale reflects the percentage of participants who exhibited significant activation in that voxel (*z* > 2.58). Brain images are registered to fsaverage space; only the left hemisphere is depicted.

## Supplemental Table 1

Model fit statistics and stepwise regressions within baseline discovery sample

| **Models** |  | **AIC** |  | **Model comparisons** | **χ^2^** | ***df*** |
| --- | --- | --- | --- | --- | --- | --- |
| Model 1 | covariates | 26961.12 |  |  |  |  |
| Model 2 | covariates + sex | 26939.31 |  | Model 2 vs. Model 1 | 24.39*** | 1 |
| Model 3 | covariates + sex + LS + CCT | 26943.79 |  | Model 3 vs. Model 2 | 6.63* | 2 |
| Model 4 | covariates + sex*CCT + LS | 26941.00 |  | Model 4 vs. Model 3 | 3.99* | 1 |
| Model 5 | covariates + sex*LS + CCT | 26940.99 |  | Model 5 vs. Model 3 | 10.00** | 1 |
| Model 6 ^P^ | covariates + sex*LS + sex*CCT | 26938.19 |  | Model 6 vs. Model 5 | 4.00* | 1 |
| Model 7 | covariates + sex*LS*CCT | 26951.07 |  | Model 7 vs. Model 6 | 0.04 | 2 |

*Note*. Model fit statistics and stepwise regressions within baseline discovery sample. Akaike Information Criterion (AIC) reflects model fit. χ^2^ value reflects ANOVA results for stepwise comparisons between models with degrees of freedom (*df*); Models 4 and 5 are not comparable, so both are compared to Model 3. Covariates include age, socioeconomic status, and stressful life events; LS = List Sorting, CCT = Cash Choice task. Asterisks indicate level of significance (uncorrected); fixed and random effects are only reported for the preferred model.

* *p* < .05; ** *p* < .01; *** *p* < .001.

^P^ specifies the model that was selected as the preferred model

## Supplemental Table 2

Preferred multilevel model in baseline sub-sample with available behavioral data in the discovery sample

|  |  |  | *BOYS*  *n = 1,862* | | | |  |  |  |  | |
| --- | --- | --- | --- | --- | --- | --- | --- | --- | --- | --- | --- |
| Fixed Effects |  | β(*SE*) | CIs | *df* | *t*-value |  | β(*SE*) | CIs | *df* | *t*-value | |
|  |  | *Unstandardized* | | | |  | *Standardized* | | | |  |
| Intercept |  | 58.9(3.76) | [51.54, 66.27] | 1778.38 | 15.68*** |  | -0.03(0.04) | [-0.11, 0.05] | 49.55 | -0.72 | |
| List Sorting |  | -0.09(0.02) | [-0.13, -0.05] | 1821.27 | -4.00*** |  | -0.09(0.02) | [-0.14, -0.05] | 1821.27 | -4.00*** | |
| Cash Choice Task |  | 0.49(0.43) | [-0.35, 1.34] | 1702.00 | 1.14 |  | 0.05(0.04) | [-0.04, 0.14] | 1702.00 | 1.14 | |
| Age (months) |  | -0.06(0.03) | [-0.11, 0.00] | 1841.29 | -1.92 |  | -0.04(0.02) | [-0.09, 0.00] | 1841.29 | -1.92 | |
| Stressful life events |  | 0.60(0.14) | [0.32, 0.87] | 1852.42 | 4.3*** |  | 0.10(0.02) | [0.05, 0.14] | 1852.42 | 4.30*** | |
| SES Composite |  | -5.58(1.37) | [-8.26, -2.91] | 1235.46 | -4.08*** |  | -0.10(0.02) | [-0.15, -0.05] | 1235.46 | -4.08*** | |
|  |  | Marginal *R^2^*: 0.033 | | | |  |  |  |  |  | |
| Random Effects |  | Variance(*SD*) | Levels |  |  |  | Variance(*SD*) |  |  |  | |
| Family:Site |  | 44.36(6.66) | 1677 |  |  |  | 0.48(0.69) |  |  |  | |
| Site |  | 0.85(0.92) | 22 |  |  |  | 0.01(0.10) |  |  |  | |
| Residual |  | 45.11(6.72) |  |  |  |  | 0.48(0.70) |  |  |  | |
|  |  | ICC: 0.50 | | | |  |  |  |  |  | |
|  |  | Conditional *R^2^*: 0.517 | | | |  |  |  |  |  | |
|  |  |  | *GIRLS*  *n = 1,852* | | |  |  |  |  |  | |
| Fixed Effects |  | β(*SE*) | CIs | *df* | *t*-value |  | β(*SE*) | CIs | *df* | *t*-value | |
|  |  | *Unstandardized* | | | |  | *Standardized* | | | | |
| Intercept |  | 51.02(3.48) | [44.22, 57.89] | 1754.63 | 14.65*** |  | 0.00(0.03) | [-0.07, 0.06] | 21.02 | -0.13 | |
| **Supplemental Table 2 Continued** | | |  |  |  |  |  |  |  |  | |
| Age (months) |  | -0.03(0.03) | [-0.09, 0.02] | 1824.78 | -1.16 |  | -0.03(0.02) | [-0.07, 0.02] | 1824.78 | -1.16 | |
| Stressful life events |  | 0.46(0.12) | [0.21, 0.70] | 1699.90 | 3.67*** |  | 0.09(0.02) | [0.04, 0.13] | 1699.90 | 3.67*** | |
| SES Composite |  | -6.11(1.23) | [-8.56, -3.70] | 1213.34 | -4.95*** |  | -0.12(0.02) | [-0.17, -0.07] | 1213.34 | -4.95*** | |
|  |  | Marginal *R^2^*: 0.024 | | | |  |  |  |  |  | |
| Random Effects |  | Variance(*SD*) | Levels |  |  |  | Variance(*SD*) |  |  |  | |
| Family:Site |  | 29.3(5.41) | 1641 |  |  |  | 0.36(0.60) |  |  |  | |
| Site |  | 0.89(0.94) | 22 |  |  |  | 0.01(0.10) |  |  |  | |
| Residual |  | 48.67(6.98) |  |  |  |  | 0.60(0.78) |  |  |  | |
|  |  | ICC: 0.38 | | | |  |  |  |  |  | |
|  |  | Conditional *R^2^*: 0.398 | | | |  |  |  |  |  | |

*Note*. Discovery sample models run separately by sex explaining variance in baseline externalizing *t*-scores using demographics (age, socioeconomic status (SES), life stressors, sex), behavioral List Sorting and Cash Choice tasks, and random intercepts (family nested within site). Unstandardized and standardized estimates are reported; beta coefficient estimates = β; *SE* = standard error; CIs = 95% confidence intervals; *df* = degrees of freedom; *SD* = standard deviation. Marginal *R²* reflects the proportion of variance explained by the fixed effects, and conditional *R²* reflects variance explained by both fixed and random effects; ICC = intraclass correlation coefficient. Asterisks indicate level of significance (uncorrected).

*** *p* < .001.

## Supplemental Table 3

Model fit statistics in baseline sub-sample with available imaging data

| **Models** |  | **AIC** |  | **Model comparisons** | **χ^2^** | ***df*** |
| --- | --- | --- | --- | --- | --- | --- |
| Model 1 | covariates_age+SES+stressful life events+fwd_ | 12138.62 |  |  |  |  |
| Model 2 ^P^ | covariates + sex | 12130.85 |  | Model 2 vs. Model 1 | 9.56** | 1 |
| Model 3a | covariates + sex + EN-back + MIDantic | 12133.13 |  | Model 3a vs. Model 2 | 2.52 | 2 |
| Model 4a | covariates + sex*MIDantic + EN-back | 12132.13 |  | Model 4a vs. Model 3a | 0.56 | 1 |
| Model 5a | covariates + sex*EN-back + MIDantic | 12132.20 |  | Model 5a vs. Model 3a | 3.55 | 1 |
| Model 6a | covariates + sex*EN-back + sex*MIDantic | 12131.11 |  | Model 6a vs. Model 5a | 0.64 | 1 |
| Model 7a | covariates + sex*EN-back*MIDantic | 12131.52 |  | Model 7a vs. Model 6a | 1.71 | 2 |
| Model 8a | covariates + sex + rsfMRI + EN-back + MIDantic | 11830.95 |  | Model 7a_rs1_ vs. Model 3a | 0.29 | 1 |
| Model 9a | covariates + sex*rsfMRI + EN-back + MIDantic | 11826.16 |  | Model 7a_rs2_ vs. Model 7a_rs1_ | 0.19 | 1 |
| Model 3f | covariates + sex + EN-back + MIDfeedback | 12135.72 |  | Model 3f vs. Model 2 | 0.32 | 2 |
| Model 4f | covariates + sex*MIDfeedback + EN-back | 12132.79 |  | Model 4f vs. Model 3f | 2.87 | 1 |
| Model 5f | covariates + sex*EN-back + MIDfeedback | 12134.86 |  | Model 5f vs. Model 3f | 3.48 | 1 |
| Model 6f | covariates + sex*EN-back + sex*MIDfeedback | 12131.81 |  | Model 6f vs. Model 5f | 2.98 | 1 |
| Model 7f | covariates + sex*EN-back*MIDfeedback | 12134.50 |  | Model 7f vs. Model 6f | 0.28 | 2 |
| Model 8f | covariates + sex + rsfMRI + EN-back + MIDfeedback | 11832.66 |  | Model 7f_rs1_ vs. Model 3f | 0.35 | 1 |
| Model 9f | covariates + sex*rsfMRI + EN-back + MIDfeedback | 11827.90 |  | Model 7f_rs2_ vs. Model 7 f_rs1_ | 0.16 | 1 |

*Note*. Model fit statistics and stepwise regressions within subset of baseline discovery sample with available task-dependent fMRI data for both tasks (*n* = 1,669); models examining subset with resting-state fMRI (rsfMRI) data (*n* = 1,628) are denoted with (_rs_). χ^2^ value reflects ANOVA results for stepwise comparisons between models with degrees of freedom (*df*); Models 4 and 5 were not comparable, so both were compared to Model 3. Covariates include framewise displacement (fwd) for emotional n-back (EN-back) and MID tasks, age, socioeconomic status (SES), stressful life events, and random intercepts (family nested within site); models were run separately for the monetary incentive delay (MID) task reward anticipation (a, MIDantic) and feedback (f, MIDfeedback) contrasts. Asterisks indicate level of significance (uncorrected).

** *p* < .01

^P^ specifies the model that was selected as the preferred model; fixed and random effects are only reported for the preferred model.

## Supplemental Table 4

Preferred multilevel model in baseline sub-sample with available imaging data in the discovery sample

| Fixed Effects |  | β(*SE*) | | CIs | | *df* | | *t*-value | |  | | β(*SE*) | | CIs | | *df* | | *t*-value | | |
| --- | --- | --- | --- | --- | --- | --- | --- | --- | --- | --- | --- | --- | --- | --- | --- | --- | --- | --- | --- | --- |
|  |  | *Unstandardized* | | | | | | | |  | | *Standardized* | | | | | | | |  |
| Intercept |  | 52.41(3.88) | [44.84, 60.07] | | 1523.76 | | 13.51*** | |  | | 0.07(0.04) | | [-0.01, 0.15] | | 58.34 | | 1.62 | |  |  |
| Sex |  | -1.41(0.46) | [-2.31, -0.52] | | 1631.08 | | -3.09** | |  | | -0.15(0.05) | | [-0.25, -0.06] | | 1631.08 | | -3.09** | |  |  |
| Age (months) |  | -0.04(0.03) | [-0.10, 0.02] | | 1620.78 | | -1.29 | |  | | -0.03(0.02) | | [-0.08, 0.02] | | 1620.78 | | -1.29 | |  |  |
| Stressful events |  | 0.48(0.14) | [0.21, 0.74] | | 1528.27 | | 3.49*** | |  | | 0.09(0.02) | | [0.04, 0.13] | | 1528.27 | | 3.49*** | |  |  |
| SES Composite |  | -6.01(1.48) | [-8.9, -3.12] | | 1158.93 | | -4.07*** | |  | | -0.1(0.03) | | [-0.15, -0.05] | | 1158.93 | | -4.07*** | |  |  |
| EN-back fwd |  | 0.50(1.03) | [-1.54, 2.52] | | 1661.30 | | 0.48 | |  | | 0.01(0.03) | | [-0.05, 0.07] | | 1661.30 | | 0.48 | |  |  |
| MID fwd |  | 0.38(1.46) | [-2.48, 3.23] | | 1660.44 | | 0.26 | |  | | 0.01(0.03) | | [-0.05, 0.07] | | 1660.44 | | 0.26 | |  |  |
|  |  | Marginal *R^2^*: 0.028 | | | | | | |  | |  | |  | |  | |  | |  |  |
| Random Effects |  | Variance(*SD*) | Levels | |  | |  | |  | | Variance(*SD*) | |  | |  | |  | |  |  |
| Family:Site |  | 19.32(4.39) | 1525 | |  | |  | |  | | 0.22(0.47) | |  | |  | |  | |  |  |
| Site |  | 0.60(0.77) | 22 | |  | |  | |  | | 0.01(0.08) | |  | |  | |  | |  |  |
| Residual |  | 63.92(7.99) |  | |  | |  | |  | | 0.74(0.86) | |  | |  | |  | |  |  |
|  |  | ICC: 0.24 | | | | | | |  | |  | |  | |  | |  | |  |  |
|  |  | Conditional *R^2^*: 0.259 | | | | | | |  | |  | |  | |  | |  | |  |  |

*Note*. Fixed and random effects for preferred multilevel model explaining variance in externalizing *t*-scores using for the baseline sub-sample who had usable task-dependent fMRI data (*n* = 1,669); covariates included age, socioeconomic status (SES), stressful life events, framewise displacement (fwd) for the emotional n-back (EN-back) and monetary incentive delay (MID) tasks, and random intercepts (family nested within site). χ^2^ value reflects ANOVA results for stepwise comparisons between models. Unstandardized and standardized estimates are reported; beta coefficient estimates = β; *SE* = standard error; CIs = 95% confidence intervals; *df* = degrees of freedom; *SD* = standard deviation. Marginal *R²* reflects the proportion of variance explained by the fixed effects, and conditional *R²* reflects variance explained by both fixed and random effects; ICC = intraclass correlation coefficient. Asterisks indicate level of significance (uncorrected).

** *p* < .01; *** *p* < .001.

## Supplemental Table 5

Model fit statistics in baseline discovery sample with available behavioral and imaging data

| **Models** |  | **AIC** |  | **Model comparisons** | **χ^2^** | ***df*** |
| --- | --- | --- | --- | --- | --- | --- |
| Model 1 | covariates | 11341.91 |  |  |  |  |
| Model 2 ^P^ | covariates + sex | 11335.64 |  | Model 2 vs. Model 1 | 8.00** | 1 |
| Model 3_b_ | covariates + sex + LS + CCT | 11343.87 |  | Model 3 vs. Model 2 | 1.06 | 2 |
| Model 4_b_ | covariates + sex*LS + CCT | 11343.85 |  | Model 4 vs. Model 3 | 6.26* | 1 |
| Model 3a_n_ | covariates + sex + EN-back + MIDantic | 11337.06 |  | Model 3a vs. Model 2 | 3.31 | 2 |
| Model 6a_n_ | covariates + sex + LS + CCT + EN-back + MIDantic | 11345.14 |  | Model 6a vs. Model 3a | 1.19 | 2 |
| Model 7a_n_ | covariates + sex + LS + CCT + EN-back +sex*MIDantic | 11343.07 |  | Model 7a vs. Model 6a | 1.57 | 1 |
| Model 8a_n_ | covariates + LS + CCT*sex + MIDantic + EN-back | 11343.73 |  | Model 8a vs. Model 6a | 1.72 | 1 |
| Model 9a_n_ | covariates + sex*EN-back + MIDantic + LS + CCT | 11344.69 |  | Model 9a vs. Model 6a | 3.02 | 1 |
| Model 10a_n_ | covariates + sex*LS + CCT + EN-back + MIDantic | 11345.05 |  | Model 10a vs. Model 4 | 3.51 | 2 |
| Model 11a_n_ | covariates + sex*EN-back + MIDantic + sex*LS + CCT | 11345.74 |  | Model 11a vs. Model 10a | 1.86 | 1 |
| Model 12a_n_ | covariates + sex*EN-back*MIDantic + sex*LS*CCT | 11350.54 |  | Model 12a vs. Model 11a | 8.13 | 6 |
| Model 3f_n_ | covariates + sex + EN-back + MIDfeedback | 11340.39 |  | Model 3f_n_ vs. Model 2 | 0.34 | 2 |
| Model 6f_n_ | covariates + sex + LS + CCT + EN-back + MIDfeedback | 11348.46 |  | Model 6f_n_ vs. Model 3f_n_ | 1.20 | 2 |
| Model 7f_n_ | covariates + sex + LS + CCT + EN-back +sex*MIDfeedback | 11344.52 |  | Model 7f_n_ vs. Model 6f_n_ | 3.82 | 1 |
| Model 8f_n_ | covariates + LS + CCT*sex + MIDfeedback + EN-back | 11347.07 |  | Model 8f_n_ vs. Model 6f_n_ | 1.70 | 1 |
| Model 9f_n_ | covariates + sex*EN-back + MIDfeedback + LS + CCT | 11348.10 |  | Model 9f_n_ vs. Model 6f_n_ | 2.93 | 1 |
| Model 10f_n_ | covariates + sex*LS + CCT + EN-back + MIDfeedback | 11348.29 |  | Model 10f_n_ vs. Model 4 | 0.64 | 2 |
| Model 11f_n_ | covariates + EN-back + sex*MIDfeedback + sex*LS + CCT | 11344.50 |  | Model 11f_n_ vs. Model 10f_n_ | 3.67 | 1 |
| Model 12f_n_ | covariates + sex*EN-back*MIDfeedback + sex*LS*CCT | 11354.08 |  | Model 12f_n_ vs. Model 11f_n_ | 7.19 | 6 |

*Note*. Model fit statistics and stepwise regressions within subset of baseline discovery sub-sample with available behavioral (_b_) and neural (_n_) data (n = 1,563). χ^2^ value reflects ANOVA results for stepwise comparisons between models with degrees of freedom (*df*); Models 4 and 5 are not comparable, so both are compared to Model 3. Covariates include age, socioeconomic status, and stressful life events, and random intercepts (family nested within site); models were run separately for the monetary incentive delay (MID) task reward anticipation (a, MIDantic) and feedback (b, MIDfeedback) contrasts. Asterisks indicate level of significance (uncorrected).

* *p* < .05; ** *p* < .01; *** *p* < .001.

^P^ specifies the model that was selected as the preferred model.

## Supplemental Table 6

Model fit statistics in two year follow up discovery sample

| **Models** |  | **AIC** |  | **Model comparisons** | **χ^2^** | ***df*** |
| --- | --- | --- | --- | --- | --- | --- |
| Model 1 | covariates | 23781.07 |  |  |  |  |
| Model 2 | covariates + sex | 23783.78 |  | Model 2 vs. Model 1 | 0.34 | 1 |
| Model 3 | covariates + sex + LS + CCT | 23781.52 |  | Model 3 vs. Model 2 | 14.46*** | 2 |
| Model 4 | covariates + sex*CCT + LS | 23783.18 |  | Model 4 vs. Model 3 | 0.02 | 1 |
| Model 5 | covariates + sex*LS + CCT | 23783.14 |  | Model 5 vs. Model 3 | 6.06* | 1 |
| Model 6 | covariates + sex*LS + sex*CCT | 23784.80 |  | Model 6 vs. Model 5 | 0.03 | 1 |
| Model 7 ^P^ | covariates + sex + LS*CCT | 23780.97 |  | Model 7 vs. Model 3 | 8.19** | 1 |
| Model 8 | covariates + sex*LS*CCT | 23789.10 |  | Model 8 vs. Model 7 | 7.51 | 2 |

*Note*. Model fit statistics and stepwise regressions within the two year follow up discovery sample. Akaike Information Criterion (AIC) reflects model fit. ANOVA results for stepwise comparisons between models with degrees of freedom (*df*); Models 4 and 5 are not comparable, so both are compared to Model 3. Covariates include baseline externalizing *t*-score, baseline age, baseline socioeconomic status, and baseline stressful life events; LS = List Sorting, CCT = Cash Choice task. Asterisks indicate level of significance (uncorrected); fixed and random effects are only reported for the preferred model.

* *p* < .05; ** *p* < .01; *** *p* < .001.

^P^ specifies the model that was selected as the preferred model

## Supplemental Table 7

Two year follow up discovery sample models by Cash Choice task response

|  |  |  | *LARGER LATER*  *n = 2,121* | | |  |  |  |  |  | |
| --- | --- | --- | --- | --- | --- | --- | --- | --- | --- | --- | --- |
| Fixed Effects |  | β(*SE*) | CIs | *df* | *t*-value |  | β(*SE*) | CIs | *df* | *t*-value | |
|  |  | *Unstandardized* | | | |  | *Standardized* | | | |  |
| Intercept |  | 14.18(3.07) | [8.17, 20.21] | 1910.88 | 4.61*** |  | 0.01(0.02) | [-0.03, 0.06] | 67.38 | 0.57 | |
| Baseline EXT |  | 0.67(0.02) | [0.64, 0.71] | 2091.99 | 41.55*** |  | 0.67(0.02) | [0.64, 0.70] | 2091.99 | 41.55*** | |
| List Sorting |  | -0.10(0.02) | [-0.14, -0.05] | 2080.47 | -4.45*** |  | -0.10(0.02) | [-0.15, -0.06] | 2080.47 | -4.45*** | |
| Sex |  | -3.76(1.53) | [-6.77, -0.78] | 2111.08 | -2.47* |  | -0.02(0.03) | [-0.08, 0.04] | 2078.55 | -0.61 | |
| Age (months) |  | 0.03(0.02) | [-0.01, 0.06] | 1978.24 | 1.45 |  | 0.02(0.02) | [-0.01, 0.05] | 1978.24 | 1.45 | |
| Stressful events |  | 0.14(0.09) | [-0.04, 0.31] | 1998.64 | 1.52 |  | 0.02(0.02) | [-0.01, 0.06] | 1998.64 | 1.52 | |
| SES Composite |  | 0.51(0.89) | [-1.23, 2.26] | 1090.14 | 0.57 |  | 0.01(0.02) | [-0.02, 0.04] | 1090.14 | 0.57 | |
| List Sorting x Sex |  | 0.07(0.03) | [0.01, 0.13] | 2111.92 | 2.40* |  | 0.08(0.03) | [0.01, 0.14] | 2111.92 | 2.40* | |
|  |  | Marginal *R^2^*: 0.466 | | | |  |  |  |  |  | |
| Random Effects |  | Variance(*SD*) | Levels |  |  |  | Variance(*SD*) |  |  |  | |
| Family:Site |  | 10.57(3.25) | 1943 |  |  |  | 0.12(0.35) |  |  |  | |
| Site |  | 0.10(0.31) | 22 |  |  |  | 0.00(0.03) |  |  |  | |
| Residual |  | 34.76(5.9) |  |  |  |  | 0.41(0.64) |  |  |  | |
|  |  | ICC: 0.23 | | | |  |  |  |  |  | |
|  |  | Conditional *R^2^*: 0.591 | | | |  |  |  |  |  | |
| **Supplemental Table 7 Continued** | | | | | |  |  |  |  |  | |
|  |  |  | *SMALLER SOONER*  *n = 1,429* | | |  |  |  |  |  | |
| Fixed Effects |  | β(*SE*) | CIs | *df* | *t*-value |  | β(*SE*) | CIs | *df* | *t*-value | |
|  |  | *Unstandardized* | | | |  | *Standardized* | | | | |
| Intercept |  | 15.00(3.66) | [7.8, 22.19] | 1423.35 | 4.09*** |  | 0.00(0.02) | [-0.04, 0.04] | 1289.41 | 0.01 | |
| Baseline EXT |  | 0.59(0.02) | [0.55, 0.63] | 1402.80 | 29.06*** |  | 0.61(0.02) | [0.57, 0.65] | 1402.80 | 29.06*** | |
| Age (months) |  | 0.00(0.02) | [-0.05, 0.04] | 1423.85 | -0.17 |  | 0.00(0.02) | [-0.04, 0.04] | 1423.85 | -0.17 | |
| Stressful events |  | 0.44(0.12) | [0.21, 0.67] | 1367.15 | 3.82*** |  | 0.08(0.02) | [0.04, 0.12] | 1367.15 | 3.82*** | |
| SES Composite |  | 2.02(1.12) | [-0.17, 4.2] | 1347.25 | 1.81 |  | 0.04(0.02) | [0.00, 0.08] | 1347.25 | 1.81 | |
|  |  | Marginal *R^2^*: 0.389 | | | |  |  |  |  |  | |
| Random Effects |  | Variance(*SD*) | Levels |  |  |  | Variance(*SD*) |  |  |  | |
| Family:Site |  | 10.44(3.23) | 1343 |  |  |  | 0.13(0.36) |  |  |  | |
| Residual |  | 39.47(6.28) |  |  |  |  | 0.48(0.69) |  |  |  | |
|  |  | ICC: 0.21 | | | |  |  |  |  |  | |
|  |  | Conditional *R^2^*: 0.517 | | | |  |  |  |  |  | |

*Note*. Discovery sample models run separately by Cash Choice task response explaining variance in externalizing (EXT) *t*-scores two years later. Covariates include age, baseline socioeconomic status (SES), baseline stressful life events, and random intercepts (family nested within site). Unstandardized and standardized estimates are reported; beta coefficient estimates = β; *SE* = standard error; CIs = 95% confidence intervals; *df* = degrees of freedom; *SD* = standard deviation. Marginal *R²* reflects the proportion of variance explained by the fixed effects, and conditional *R²* reflects variance explained by both fixed and random effects; ICC = intraclass correlation coefficient. Asterisks indicate level of significance (uncorrected); fixed and random effects only reported for the preferred model. The smaller sooner models would not converge when site was included as a random intercept in the model, therefore was left out.

* *p* < .05; *** *p* < .001

^P^ specifies the model that was selected as the preferred model

## Supplemental Table 8

Model fit statistics in two year follow up discovery sub-sample with available imaging data

| **Models** |  | **AIC** |  | **Model comparisons** | **χ^2^** | ***df*** |
| --- | --- | --- | --- | --- | --- | --- |
| Model 1 ^P^ | covariates_age+SES+stressful life events+fwd_ | 10754.06 |  |  |  |  |
| Model 2 | covariates + sex | 10756.31 |  | Model 2 vs. Model 1 | 0.00 | 1 |
| Model 3a | covariates + sex + EN-back + MIDantic | 10761.00 |  | Model 3a vs. Model 2 | 1.16 | 2 |
| Model 4a | covariates + sex*MIDantic + EN-back | 10761.07 |  | Model 4a vs. Model 3a | 0.00 | 1 |
| Model 5a | covariates + sex*EN-back + MIDantic | 10763.14 |  | Model 5a vs. Model 3a | 0.99 | 1 |
| Model 6a | covariates + sex*EN-back + sex*MIDantic | 10763.19 |  | Model 6a vs. Model 5a | 0.01 | 1 |
| Model 7a | covariates + sex*EN-back*MIDantic | 10759.49 |  | Model 7a vs. Model 6a | 6.92* | 2 |
| Model 7a_rs1_ | covariates + sex + rsfMRI + EN-back + MIDantic | 10477.48 |  | Model 8a vs. Model 3a | 1.17 | 1 |
| Model 7a_rs2_ | covariates + sex*rsfMRI + EN-back + MIDantic | 10473.27 |  | Model 9a vs. Model 8a | 0.15 | 1 |
| Model 3f | covariates + sex + EN-back + MIDfeedback | 10761.72 |  | Model 3b vs. Model 2 | 0.85 | 2 |
| Model 4f | covariates + sex*MIDfeedback + EN-back | 10758.53 |  | Model 4b vs. Model 3b | 3.67 | 1 |
| Model 5f | covariates + sex*EN-back + MIDfeedback | 10763.84 |  | Model 5b vs. Model 3b | 1.00 | 1 |
| Model 6f | covariates + sex*EN-back + sex*MIDfeedback | 10760.70 |  | Model 6b vs. Model 5b | 3.62 | 1 |
| Model 7f | covariates + sex*EN-back*MIDfeedback | 10763.90 |  | Model 7b vs. Model 6b | 0.83 | 2 |
| Model 7f_rs1_ | covariates + sex + rsfMRI + EN-back + MIDfeedback | 10477.72 |  | Model 8b vs. Model 3b | 1.16 | 1 |
| Model 7f_rs2_ | covariates + sex*rsfMRI + EN-back + MIDfeedback | 10473.50 |  | Model 9b vs. Model 8b | 0.15 | 1 |

*Note*. Model fit statistics and stepwise regressions within subset of two year follow up discovery sample with available data for both fMRI tasks (*n* = 1,605); Akaike Information Criterion (AIC) reflects model fit; models that included the subset of participants who also had available resting-state fMRI (rsfMRI) data (*n* = 1,565) were denoted using (_rs_). χ^2^ value reflects ANOVA results for stepwise comparisons between models with degrees of freedom (*df*); Models 4 and 5 were not comparable, so both were compared to Model 3. Covariates include framewise displacement (fwd) for emotional n-back (EN-back) and MID tasks, age, socioeconomic status (SES), stressful life events, and random intercepts (family nested within site); models were run separately for the monetary incentive delay (MID) task reward anticipation (a, MIDantic) and feedback (f, MIDfeedback) contrasts. Asterisks indicate level of significance (uncorrected).

** *p* < .01

^P^ specifies the model that was selected as the preferred model.

## Supplemental Table 9

Model fit statistics in longitudinal discovery sub-sample with available behavioral and imaging data

| **Models** |  | **AIC** |  | **Model comparisons** | **χ^2^** |
| --- | --- | --- | --- | --- | --- |
| Model 1 | covariates | 10084.17 |  |  |  |
| Model 2 | covariates + sex | 10086.29 |  | Model 2 vs. Model 1 | 0.07 |
| Model 3 | covariates + sex + LS + CCT | 10091.42 |  | Model 3 vs. Model 2 | 5.23 |
| Model 4 ^P^ | covariates + sex*LS + CCT | 10092.53 |  | Model 4 vs. Model 3 | 5.68* |
| Model 5 | covariates + sex + EN-back + MIDantic | 10089.88 |  | Model 5a vs. Model 2 | 2.12 |
| Model 6 | covariates + sex + LS + CCT + EN-back + MIDantic | 10095.33 |  | Model 6a vs. Model 3a | 4.89 |
| Model 7 | covariates + sex + LS + CCT + EN-back +sex*MIDantic | 10095.31 |  | Model 7a vs. Model 6a | 0.02 |
| Model 8 | covariates + LS + CCT*sex + MIDantic + EN-back | 10095.33 |  | Model 8a vs. Model 6a | 0.82 |
| Model 9 | covariates + sex*EN-back + MIDantic + LS + CCT | 10098.07 |  | Model 9a vs. Model 6a | 0.32 |
| Model 10 | covariates + sex*LS + CCT + EN-back + MIDantic | 10096.70 |  | Model 10a vs. Model 4 | 1.53 |
| Model 11 | covariates + sex*EN-back + MIDantic + sex*LS + CCT | 10098.86 |  | Model 11a vs. Model 10a | 0.87 |
| Model 12 | covariates + sex*EN-back*MIDantic + sex*LS*CCT | 10101.61 |  | Model 12a vs. Model 11a | 13.33* |
| Model 5 | covariates + sex + EN-back + MIDfeedback | 10090.73 |  | Model 5b vs. Model 2 | 1.68 |
| Model 6 | covariates + sex + LS + CCT + EN-back + MIDfeedback | 10096.18 |  | Model 6b vs. Model 3b | 4.89 |
| Model 7 | covariates + sex + LS + CCT + EN-back +sex*MIDfeedback | 10094.87 |  | Model 7b vs. Model 6b | 1.71 |
| Model 8 | covariates + LS + CCT*sex + MIDfeedback + EN-back | 10096.21 |  | Model 8b vs. Model 6b | 0.79 |
| Model 9 | covariates + sex*EN-back + MIDfeedback + LS + CCT | 10098.91 |  | Model 9b vs. Model 6b | 0.33 |
| Model 10 | covariates + sex*LS + CCT + EN-back + MIDfeedback | 10097.54 |  | Model 10b vs. Model 4 | 1.09 |
| Model 11 | covariates + EN-back + sex*MIDfeedback + sex*LS + CCT | 10096.37 |  | Model 11b vs. Model 10b | 1.58 |
| Model 12 | covariates + sex*EN-back*MIDfeedback + sex*LS*CCT | 10108.73 |  | Model 12b vs. Model 11b | 7.53 |

*Note*. Model fit statistics and stepwise regressions within subset of longitudinal discovery sub-sample with available behavioral and imaging data (*n* = 1,504). Akaike Information Criterion (AIC) reflects model fit. χ^2^ value reflects ANOVA results for stepwise comparisons between models with degrees of freedom (*df*); Models 7-9 are not comparable, so all are compared to Model 6. Covariates include baseline externalizing t-score, age, socioeconomic status, stressful life events, and framewise displacement for each of the fMRI tasks, and random intercepts (family nested within site); models were run separately for the monetary incentive delay (MID) task reward anticipation (a, MIDantic) and feedback (b, MIDfeedback) contrasts. Asterisks indicate level of significance (uncorrected).

* *p* < .05; ** *p* < .01; *** *p* < .001

^P^ specifies the model that was selected as the preferred model.

## Supplemental Table 10

Baseline models in the discovery and replication samples controlling for general cognition

|  |  |  | *Discovery sample*  *n* = 3,714 | | |  | |  | | |  |  |  | |
| --- | --- | --- | --- | --- | --- | --- | --- | --- | --- | --- | --- | --- | --- | --- |
| Fixed Effects |  | β(*SE*) | CIs | *df* | *t*-value | |  | | β(*SE*) | CIs | | *df* | | *t*-value |
|  |  | *Unstandardized* | | | |  | | *Standardized* | | | | | | |
| Intercept |  | 58.11(2.65) | [52.94, 63.31] | 3383.52 | 21.95*** | |  | | 0.08(0.04) | [0.01, 0.15] | | 60.01 | | 2.20* |
| General cognition |  | -0.07(0.03) | [-0.12, -0.02] | 3688.66 | -2.88** | |  | | -0.05(0.02) | [-0.08, -0.02] | | 3688.66 | | -2.88** |
| List Sorting |  | -0.02(0.02) | [-0.05, 0.01] | 3642.71 | -1.42 | |  | | -0.02(0.02) | [-0.06, 0.01] | | 3642.71 | | -1.42 |
| Cash Choice |  | -0.10(0.3) | [-0.68, 0.49] | 3482.12 | -0.33 | |  | | -0.01(0.03) | [-0.07, 0.05] | | 3482.12 | | -0.33 |
| Sex |  | -1.46(0.3) | [-2.06, -0.87] | 3680.60 | -4.81*** | |  | | -0.16(0.03) | [-0.22, -0.09] | | 3680.6 | | -4.81*** |
| Age (months) |  | -0.04(0.02) | [-0.08, -0.01] | 3505.73 | -2.23* | |  | | -0.04(0.02) | [-0.07, 0.00] | | 3505.73 | | -2.23* |
| Stressful events |  | 0.53(0.09) | [0.35, 0.71] | 3586.15 | 5.69*** | |  | | 0.09(0.02) | [0.06, 0.13] | | 3586.15 | | 5.69*** |
| SES Composite |  | -5.43(0.94) | [-7.29, -3.58] | 2315.84 | -5.75*** | |  | | -0.1(0.02) | [-0.14, -0.07] | | 2315.84 | | -5.75*** |
|  |  | Marginal *R^2^*: 0.033 | | | | |  | |  |  | |  | |  |
| Random Effects |  | Variance(*SD*) | Levels |  |  | |  | | Variance(*SD*) |  | |  | |  |
| Family:Site |  | 34.20(5.85) | 3190 |  |  | |  | | 0.39(0.63) |  | |  | |  |
| Site |  | 0.89(0.95) | 22 |  |  | |  | | 0.01(0.1) |  | |  | |  |
| Residual |  | 49.53(7.04) |  |  |  | |  | | 0.57(0.75) |  | |  | |  |
|  |  | ICC: 0.41 | | | | |  | |  |  | |  | |  |
|  |  | Conditional *R^2^*: 0.434 | | | | |  | |  |  | |  | |  |
|  |  |  | *Replication sample*  *n* = 3,734 | | |  | |  | | |  |  |  | |
| Fixed Effects |  | β(*SE*) | CIs | *df* | *t*-value | |  | | β(*SE*) | CIs | | *df* | | *t*-value |
|  |  | *Unstandardized* | | | |  | | *Standardized* | | | | | | |
| Intercept |  | 54.76(2.65) | [49.59, 59.97] | 3252.4 | 20.69*** | |  | | 0.06(0.04) | [-0.01, 0.13] | | 61.82 | | 1.57 |
| **Supplemental Table 10 Continued** | | | |  |  | |  | |  |  | |  | |  |
| General cognition |  | -0.07(0.03) | [-0.12, -0.02] | 3689.63 | -2.78** | |  | | -0.05(0.02) | [-0.08, -0.01] | | 3689.63 | | -2.78** |
| List Sorting |  | -0.04(0.02) | [-0.07, -0.01] | 3654.81 | -2.54* | |  | | -0.04(0.02) | [-0.08, -0.01] | | 3654.81 | | -2.54* |
| Cash Choice |  | -0.27(0.30) | [-0.86, 0.32] | 3404.02 | -0.90 | |  | | -0.03(0.03) | [-0.09, 0.03] | | 3404.02 | | -0.90 |
| Sex |  | -0.89(0.31) | [-1.50, -0.29] | 3714.27 | -2.90** | |  | | -0.09(0.03) | [-0.16, -0.03] | | 3714.27 | | -2.90** |
| Age (months) |  | -0.01(0.02) | [-0.05, 0.03] | 3341.4 | -0.35 | |  | | -0.01(0.02) | [-0.04, 0.03] | | 3341.40 | | -0.35 |
| Stressful events |  | 0.55(0.09) | [0.37, 0.73] | 3641.64 | 6.04*** | |  | | 0.10(0.02) | [0.07, 0.13] | | 3641.64 | | 6.04*** |
| SES Composite |  | -6.16(0.97) | [-8.06, -4.27] | 2453.08 | -6.36*** | |  | | -0.11(0.02) | [-0.15, -0.08] | | 2453.08 | | -6.36*** |
|  |  | Marginal *R^2^*: 0.034 | | | | |  | |  |  | |  | |  |
| Random Effects |  | Variance(*SD*) | Levels |  |  | |  | | Variance(*SD*) |  | |  | |  |
| Family:Site |  | 38.25(6.18) | 3214 |  |  | |  | | 0.42(0.65) |  | |  | |  |
| Site |  | 1.06(1.03) | 22 |  |  | |  | | 0.01(0.11) |  | |  | |  |
| Residual |  | 48.55(6.97) |  |  |  | |  | | 0.53(0.73) |  | |  | |  |
|  |  | ICC: 0.45 | | | | |  | |  |  | |  | |  |
|  |  | Conditional *R^2^*: 0.466 | | | | |  | |  |  | |  | |  |

*Note*. Baseline discovery and replication sample fixed and random effects for preferred multilevel model explaining variance in baseline externalizing *t*-scores using demographics (age, socioeconomic status (SES), life stressors, sex), general cognition (NIH Toolbox composite score, excluding List Sorting task), List Sorting task, Cash Choice task, and random intercepts (family nested within site). Unstandardized and standardized estimates are reported; beta coefficient estimates = β; *SE* = standard error; CIs = 95% confidence intervals; *df* = degrees of freedom; *SD* = standard deviation. Marginal *R²* reflects the proportion of variance explained by the fixed effects, and conditional *R²* reflects variance explained by both fixed and random effects; ICC = intraclass correlation coefficient. Asterisks indicate level of significance (uncorrected).

* *p* < .05; ** *p* < .01; *** *p* < .001

## Supplemental Table 11

Baseline models in the discovery and replication samples controlling for internalizing behaviors

|  |  |  | *Discovery sample*  *n* = 3,714 | | | |  | |  | |  |  |  |  |
| --- | --- | --- | --- | --- | --- | --- | --- | --- | --- | --- | --- | --- | --- | --- |
| Fixed Effects |  | β(*SE*) | CIs | *df* | *t*-value |  | | β(*SE*) | | CIs | | *df* | *t*-value |  |
|  |  | *Unstandardized* | | | | |  | | *Standardized* | | | | | |
| Intercept |  | 32.81(2.30) | [28.28, 37.31] | 3236.18 | 14.27*** |  | | 0.06(0.03) | | [0.00, 0.11] | | 104.47 | 2.09* |  |
| Internalizing |  | 0.49(0.01) | [0.46, 0.51] | 3519.10 | 37.28*** |  | | 0.52(0.01) | | [0.49, 0.54] | | 3519.10 | 37.28*** |  |
| List Sorting |  | -0.03(0.01) | [-0.06, 0.00] | 3669.72 | -2.33* |  | | -0.03(0.01) | | [-0.06, -0.01] | | 3669.72 | -2.33* |  |
| Cash Choice |  | -0.08(0.26) | [-0.59, 0.43] | 3668.87 | -0.32 |  | | -0.01(0.03) | | [-0.06, 0.05] | | 3668.87 | -0.32 |  |
| Sex |  | -0.94(0.26) | [-1.45, -0.43] | 3594.47 | -3.63*** |  | | -0.10(0.03) | | [-0.15, -0.05] | | 3594.47 | -3.63*** |  |
| Age (months) |  | -0.05(0.02) | [-0.09, -0.02] | 3310.96 | -3.09** |  | | -0.04(0.01) | | [-0.07, -0.02] | | 3310.96 | -3.09** |  |
| Stressful events |  | 0.28(0.08) | [0.12, 0.43] | 3178.75 | 3.52*** |  | | 0.05(0.01) | | [0.02, 0.08] | | 3178.75 | 3.52*** |  |
| SES Composite |  | -5.08(0.77) | [-6.59, -3.59] | 1170.85 | -6.63*** |  | | -0.09(0.01) | | [-0.12, -0.07] | | 1170.85 | -6.63*** |  |
|  |  | Marginal *R^2^*: 0.300 | | | |  | |  | |  | |  |  |  |
| Random Effects |  | Variance(*SD*) | Levels |  |  |  | | Variance(*SD*) | |  | |  |  |  |
| Family:Site |  | 12.04(3.47) | 3190 |  |  |  | | 0.14(0.37) | |  | |  |  |  |
| Site |  | 0.10(0.32) | 22 |  |  |  | | 0.00(0.03) | |  | |  |  |  |
| Residual |  | 48.59(6.97) |  |  |  |  | | 0.56(0.75) | |  | |  |  |  |
|  |  | ICC: 0.20 | | | |  | |  | |  | |  |  |  |
|  |  | Conditional *R^2^*: 0.440 | | | |  | |  | |  | |  |  |  |
|  |  |  | *Replication sample*  *n* = 3,734 | | | |  | |  | |  |  |  |  |
| Fixed Effects |  | β(*SE*) | CIs | *df* | *t*-value |  | | β(*SE*) | | CIs | | *df* | *t*-value |  |
|  |  | *Unstandardized* | | | | |  | | *Standardized* | | | | | |
| Intercept |  | 31.85(2.28) | [27.36, 36.32] | 3213.02 | 13.95*** |  | | 0.02(0.03) | | [-0.04, 0.07] | | 108.13 | 0.65 |  |
| **Supplemental Table 11 Continued** | | |  |  |  |  | |  | |  | |  |  |  |
| Internalizing |  | 0.49(0.01) | [0.46, 0.51] | 3571.79 | 36.63*** |  | | 0.51(0.01) | | [0.49, 0.54] | | 3571.79 | 36.63*** |  |
| List Sorting |  | -0.05(0.01) | [-0.08, -0.03] | 3724.27 | -3.84*** |  | | -0.05(0.01) | | [-0.08, -0.03] | | 3724.27 | -3.84*** |  |
| Cash Choice |  | -0.22(0.26) | [-0.73, 0.30] | 3614.82 | -0.83 |  | | -0.02(0.03) | | [-0.08, 0.03] | | 3614.82 | -0.83 |  |
| Sex |  | -0.12(0.27) | [-0.64, 0.40] | 3671.60 | -0.44 |  | | -0.01(0.03) | | [-0.07, 0.04] | | 3671.60 | -0.44 |  |
| Age (months) |  | -0.04(0.02) | [-0.07, 0] | 3394.68 | -2.10* |  | | -0.03(0.01) | | [-0.06, 0] | | 3394.68 | -2.10* |  |
| Stressful events |  | 0.22(0.08) | [0.07, 0.38] | 3389.55 | 2.87** |  | | 0.04(0.01) | | [0.01, 0.07] | | 3389.55 | 2.87** |  |
| SES Composite |  | -5.34(0.80) | [-6.9, -3.78] | 1438.04 | -6.69*** |  | | -0.10(0.01) | | [-0.13, -0.07] | | 1438.04 | -6.69*** |  |
|  |  | Marginal *R^2^*: 0.294 | | | |  | |  | |  | |  |  |  |
| Random Effects |  | Variance(*SD*) | Levels |  |  |  | | Variance(*SD*) | |  | |  |  |  |
| Family:Site |  | 17.8(4.22) | 3214 |  |  |  | | 0.2(0.44) | |  | |  |  |  |
| Site |  | 0.20(0.44) | 22 |  |  |  | | 0.00(0.05) | |  | |  |  |  |
| Residual |  | 45.63(6.76) |  |  |  |  | | 0.5(0.71) | |  | |  |  |  |
|  |  | ICC: 0.28 | | | |  | |  | |  | |  |  |  |
|  |  | Conditional *R^2^*: 0.494 | | | |  | |  | |  | |  |  |  |

*Note*. Discovery and replication sample fixed and random effects for multilevel model explaining variance in baseline externalizing *t*-scores controlling for internalizing *t*-scores. Unstandardized and standardized estimates are reported; beta coefficient estimates = β; *SE* = standard error; CIs = 95% confidence intervals; *df* = degrees of freedom; *SD* = standard deviation. Marginal *R²* reflects the proportion of variance explained by the fixed effects, and conditional *R²* reflects variance explained by both fixed and random effects; ICC = intraclass correlation coefficient. Asterisks indicate level of significance (uncorrected).

* *p* < .05; ** *p* < .01; *** *p* < .001

## Supplemental Table 12

Models in the two year follow up discovery and replication samples controlling for internalizing behaviors

|  |  |  | *Discovery sample n* = 3,550 | | | | | |  | | | |  | | | |  | |  | |  | |  |  |
| --- | --- | --- | --- | --- | --- | --- | --- | --- | --- | --- | --- | --- | --- | --- | --- | --- | --- | --- | --- | --- | --- | --- | --- | --- |
| Fixed Effects |  | β(*SE*) | CIs | | *df* | | *t*-value | | |  | | | | β(*SE*) | | | | CIs | | *df* | | *t*-value | |  |
|  |  | *Unstandardized* | | | | | | | | |  | | | | *Standardized* | | | | | | | | | |
| Intercept |  | 13.48(2.36) | [8.86, 18.09] | 2995.78 | | 5.71*** | |  | | | | -0.02(0.02) | | | | [-0.07, 0.02] | | | 176.42 | | -1.05 | |  |  |
| Baseline Externalizing |  | 0.63(0.01) | [0.6, 0.66] | 3532.67 | | 42.54*** | |  | | | | 0.64(0.02) | | | | [0.61, 0.67] | | | 3532.67 | | 42.54*** | |  |  |
| Baseline Internalizing |  | 0.01(0.01) | [-0.02, 0.04] | 3475.94 | | 0.79 | |  | | | | 0.01(0.01) | | | | [-0.02, 0.04] | | | 3475.94 | | 0.79 | |  |  |
| List Sorting |  | -0.03(0.01) | [-0.06, -0.01] | 3490.04 | | -2.91** | |  | | | | -0.04(0.01) | | | | [-0.06, -0.01] | | | 3490.04 | | -2.91** | |  |  |
| Cash Choice Task |  | 0.57(0.23) | [0.11, 1.03] | 3467.13 | | 2.44* | |  | | | | 0.06(0.03) | | | | [0.01, 0.11] | | | 3467.13 | | 2.44* | |  |  |
| Sex |  | -0.18(0.24) | [-0.64, 0.28] | 3477.34 | | -0.77 | |  | | | | -0.02(0.03) | | | | [-0.07, 0.03] | | | 3477.34 | | -0.77 | |  |  |
| Age (months) |  | 0.01(0.01) | [-0.02, 0.04] | 3104.22 | | 0.82 | |  | | | | 0.01(0.01) | | | | [-0.01, 0.04] | | | 3104.21 | | 0.82 | |  |  |
| Stressful life events |  | 0.22(0.07) | [0.08, 0.36] | 3193.78 | | 3.1** | |  | | | | 0.04(0.01) | | | | [0.01, 0.07] | | | 3193.78 | | 3.1** | |  |  |
| SES Composite |  | 0.97(0.71) | [-0.41, 2.37] | 1320.35 | | 1.37 | |  | | | | 0.02(0.01) | | | | [-0.01, 0.04] | | | 1320.35 | | 1.37 | |  |  |
|  |  | Marginal *R^2^*: 0.429 | | | | | |  | | | |  | | | |  | | |  | |  | |  |  |
| Random Effects |  | Variance(*SD*) | Levels |  | |  | |  | | | | Variance(*SD*) | | | |  | | |  | |  | |  |  |
| Family:Site |  | 13.55(3.68) | 3057 |  | |  | |  | | | | 0.16(0.4) | | | |  | | |  | |  | |  |  |
| Site |  | 0.03(0.17) | 22 |  | |  | |  | | | | 0(0.02) | | | |  | | |  | |  | |  |  |
| Residual |  | 33.96(5.83) |  |  | |  | |  | | | | 0.4(0.64) | | | |  | | |  | |  | |  |  |
|  |  | ICC: 0.29 | | | | | |  | | | |  | | | |  | | |  | |  | |  |  |
|  |  | Conditional *R^2^*: 0.592 | | | | | |  | | | |  | | | |  | | |  | |  | |  |  |
|  |  |  | *Replication sample n* = 3,577 | | | | | |  | | | |  | | | |  | |  | |  | |  |  |
| Fixed Effects |  | β(*SE*) | CIs | | *df* | | *t*-value | | |  | | | | β(*SE*) | | | | CIs | | *df* | | *t*-value | |  |
|  |  | *Unstandardized* | | | | | | | | |  | | | | *Standardized* | | | | | | | | | |
| **Supplemental Table 12 Continued** | | |  | |  | |  | | |  | | | |  | | | |  | |  | |  | |  |
| Intercept |  | 11.99(2.32) | [7.46, 16.61] | | 3086.44 | | 5.16*** | | |  | | | | 0(0.03) | | | | [-0.06, 0.05] | | 68.34 | | -0.14 | |  |
| Baseline Externalizing |  | 0.60(0.01) | [0.57, 0.63] | | 3566.38 | | 41.05*** | | |  | | | | 0.62(0.02) | | | | [0.59, 0.65] | | 3566.38 | | 41.05*** | |  |
| Baseline Internalizing |  | 0.02(0.01) | [-0.01, 0.05] | | 3563.40 | | 1.57 | | |  | | | | 0.02(0.02) | | | | [-0.01, 0.05] | | 3563.40 | | 1.57 | |  |
| List Sorting |  | -0.01(0.01) | [-0.03, 0.01] | | 3561.10 | | -0.96 | | |  | | | | -0.01(0.01) | | | | [-0.04, 0.01] | | 3561.10 | | -0.96 | |  |
| Cash Choice Task |  | 0.13(0.23) | [-0.33, 0.59] | | 3444.00 | | 0.54 | | |  | | | | 0.01(0.03) | | | | [-0.04, 0.06] | | 3444.00 | | 0.54 | |  |
| Sex |  | 0.00(0.24) | [-0.46, 0.47] | | 3518.79 | | 0.02 | | |  | | | | 0(0.03) | | | | [-0.05, 0.05] | | 3518.79 | | 0.02 | |  |
| Age (months) |  | 0.03(0.01) | [0.00, 0.05] | | 3338.84 | | 1.73 | | |  | | | | 0.02(0.01) | | | | [0.00, 0.05] | | 3338.84 | | 1.73 | |  |
| Stressful life events |  | 0.31(0.07) | [0.17, 0.45] | | 3398.39 | | 4.41*** | | |  | | | | 0.06(0.01) | | | | [0.03, 0.08] | | 3398.39 | | 4.41*** | |  |
| SES Composite |  | 0.07(0.74) | [-1.38, 1.51] | | 1879.78 | | 0.09 | | |  | | | | 0(0.01) | | | | [-0.03, 0.03] | | 1879.78 | | 0.09 | |  |
|  |  | Marginal *R^2^*: 0.419 | | | | | | | |  | | | |  | | | |  | |  | |  | |  |
| Random Effects |  | Variance(SD) | Levels | |  | |  | | |  | | | | Variance(SD) | | | |  | |  | |  | |  |
| Family:Site |  | 14.4(3.79) | 3075 | |  | |  | | |  | | | | 0.17(0.41) | | | |  | |  | |  | |  |
| Site |  | 0.36(0.6) | 22 | |  | |  | | |  | | | | 0.00(0.07) | | | |  | |  | |  | |  |
| Residual |  | 33.8(5.81) |  | |  | |  | | |  | | | | 0.4(0.63) | | | |  | |  | |  | |  |
|  |  | ICC: 0.30 | | | | | | | |  | | | |  | | | |  | |  | |  | |  |
|  |  | Conditional *R^2^*: 0.595 | | | | | | | |  | | | |  | | | |  | |  | |  | |  |

*Note*. Two year follow up discovery and replication sample effects for multilevel model explaining variance in externalizing *t*-scores two years later, controlling for baseline externalizing and baseline internalizing *t*-scores. Unstandardized and standardized estimates are reported; beta coefficient estimates = β; *SE* = standard error; *df* = degrees of freedom; *SD* = standard deviation. Marginal *R²* reflects the proportion of variance explained by the fixed effects, and conditional *R²* reflects variance explained by both fixed and random effects; ICC = intraclass correlation coefficient. Asterisks indicate level of significance (uncorrected).

* *p* < .05; ** *p* < .01; *** *p* < .001

## Supplemental Figure 2

Q-Q plots for preferred model


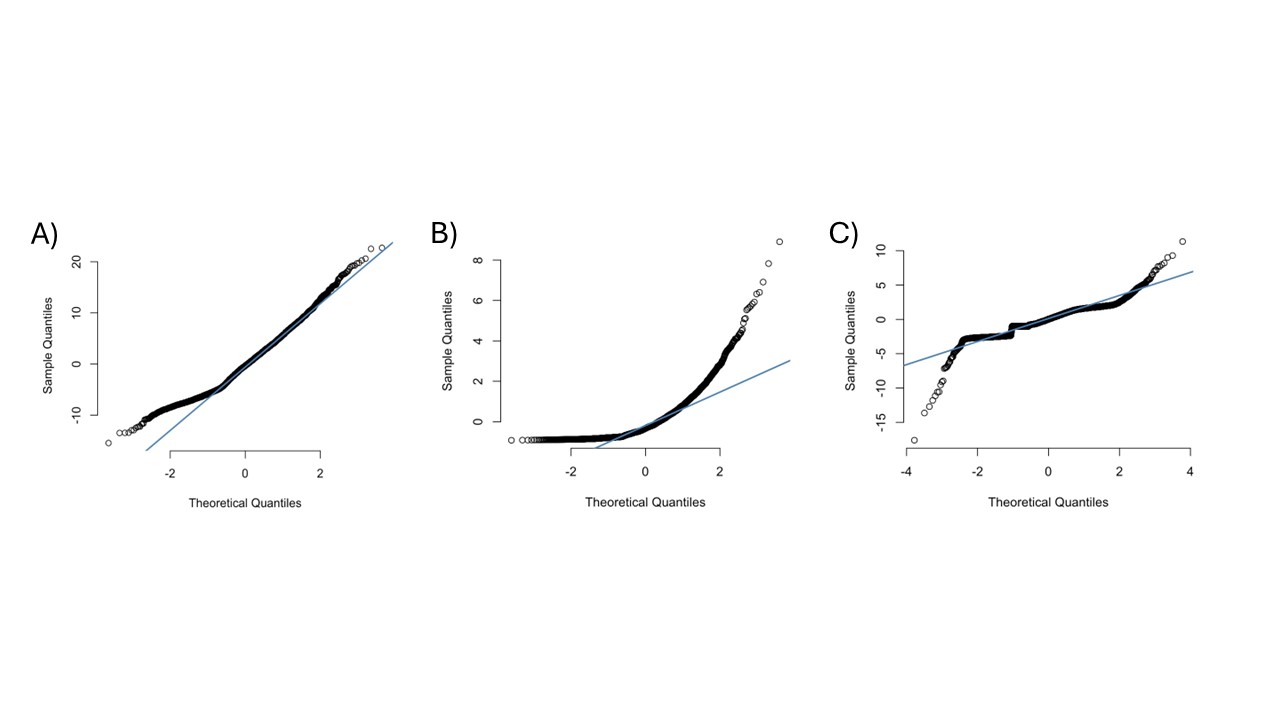


*Note*. Normal Q-Q plots for A) linear mixed effects, B) zero-inflated negative binomial, and C) negative binomial models for the preferred model including covariates and behavioral measures of working memory (List Sorting task) and reward processing (i.e., Cash Choice task).

## Supplemental Table 13

Fit statistics for baseline negative binomial and zero-inflated negative binomial models

| *Zero-inflated negative binomials models* | | | | | |
| --- | --- | --- | --- | --- | --- |
| Models |  | AIC |  | Model comparisons | Likelihood Ratio |
| Model 1 | covariates | 15602.52 |  |  |  |
| Model 2 | covariates + sex | 15552.65 |  | Model 2 vs. Model 1 | 53.87*** |
| Model 3 | covariates + sex + LS + CCT | 15555.79 |  | Model 3 vs. Model 2 | 4.86 |
| Model 4 | covariates + sex*LS + CCT | 15549.30 |  | Model 4 vs. Model 3 | 10.49** |
| Model 5 | covariates + sex*CCT + LS | 15557.62 |  | Model 5 vs. Model 3 | 2.17 |
| Model 6 | covariates + sex*LS*CCT | 15556.96 |  | Model 6 vs. Model 5 | 4.34 |
| *Negative binomials models* | | | | | |
| Models |  | AIC |  | Model | χ^2^ |
| Model 1 | covariates | 15611.99 |  |  |  |
| Model 2 | covariates + sex | 15568.40 |  | Model 2 vs. Model 1 | 45.59*** |
| Model 3 | covariates + sex + LS + CCT | 15568.12 |  | Model 3 vs. Model 2 | -4.29 |
| Model 4 | covariates + sex*LS + CCT | 15563.02 |  | Model 4 vs. Model 3 | -7.09** |
| Model 5 | covariates + sex*CCT + LS | 15568.06 |  | Model 5 vs. Model 3 | -2.06 |
| Model 6 | covariates + sex*LS*CCT | 15566.82 |  | Model 6 vs. Model 5 | -2.20 |

*Note*. Fit statistics for negative binomial and zero-inflated negative binomial models at baseline in the discovery sample for behavioral models including covariates (age, socioeconomic status, and stressful life events), List Sorting (LS), and Cash Choice task (CCT) measures.

## Supplemental Table 14

Fit statistics for longitudinal negative binomial and zero-inflated negative binomial models

| *Zero-inflated negative binomials models* | | | | | |
| --- | --- | --- | --- | --- | --- |
| Models |  | AIC |  | Model comparisons | Likelihood Ratio |
| Model 1 | covariates | 12772.18 |  |  |  |
| Model 2 | covariates + sex | 12772.72 |  | Model 2 vs. Model 1 | 3.46 |
| Model 3 | covariates + sex + LS + CCT | 12770.31 |  | Model 3 vs. Model 2 | 10.41* |
| Model 4 | covariates + sex*LS + CCT | 12765.52 |  | Model 4 vs. Model 3 | 8.79* |
| Model 5 | covariates + sex*CCT + LS | 12773.14 |  | Model 5 vs. Model 3 | 1.17 |
| Model 6 | covariates + sex*LS*CCT | 12768.60 |  | Model 6 vs. Model 5 | 8.92 |
| *Negative binomials models* | | | | | |
| Models |  | AIC |  | Model | χ^2^ |
| Model 1 | covariates | 13168.47 |  |  |  |
| Model 2 | covariates + sex | 13167.96 |  | Model 2 vs. Model 1 | 2.51 |
| Model 3 | covariates + sex + LS + CCT | 13162.10 |  | Model 3 vs. Model 2 | -9.86** |
| Model 4 | covariates + sex*LS + CCT | 13155.40 |  | Model 4 vs. Model 3 | -8.71** |
| Model 5 | covariates + sex*CCT + LS | 13163.94 |  | Model 5 vs. Model 3 | -0.17 |
| Model 6 | covariates + sex*LS*CCT | 13155.39 |  | Model 6 vs. Model 5 | -6.01 |

*Note*. Fit statistics for longitudinal negative binomial and zero-inflated negative binomial models at baseline in the discovery sample for behavioral models including covariates (baseline externalizing, age, socioeconomic status, and stressful life events), List Sorting (LS), and Cash Choice task (CCT) measures.

## Supplemental Table 15

Bootstrapped ridge regressions in the discovery sample

|  | *Baseline* |  |
| --- | --- | --- |
| **Effects** | **Mean(*SD*)** | **CIs** |
| Intercept | 51.355(2.245) | [51.216,51.494] |
| List Sorting | -0.036(0.027) | [-0.038,-0.034] |
| Sex | -1.275(0.560) | [-1.309,-1.240] |
| Stressful life events | 0.463(0.183) | [0.451,0.474] |
| SES | -4.814(1.778) | [-4.925,-4.704] |
| Deviance ratio | 0.034(0.012) | [0.033 – 0.034] |
|  | *Two year follow up* | |
| **Effects** | **Mean(*SD*)** | **CIs** |
| Intercept | 16.004(0.976) | [15.943,16.064] |
| Baseline Externalizing | 0.608(0.022) | [0.607,0.610] |
| Stressful life events | 0.195(0.129) | [0.188,0.203] |
| Deviance ratio | 0.434(0.026) | [0.432,0.436] |

*Note*. Mean, standard deviation (*SD*), and 95% confidence intervals (CIs) for 1,000 bootstrapped, 5-fold cross-validated ridge regressions within the discovery sample.
